# Genome-wide identification, phylogenetic investigation and abiotic stress responses analysis of the *PP2C* gene family in litchi (*Litchi chinensis* Sonn.)

**DOI:** 10.3389/fpls.2025.1547526

**Published:** 2025-04-15

**Authors:** Jie Yang, Rong Chen, Wei Liu, Chao Fan

**Affiliations:** Guangdong Provincial Key Laboratory of Science and Technology Research on Fruit Tree, Key Laboratory of South Subtropical Fruit Biology and Genetic Resource Utilization, Ministry of Agriculture and Rural Affairs, Institute of Fruit Tree Research, Guangdong Academy of Agricultural Sciences, Guangzhou, China

**Keywords:** PP2C gene family, litchi, phylogenetic tree, abiotic stresses, expression pattern, physiological biochemistry

## Abstract

As an important regulatory protein phosphatase in the abscisic acid (ABA) signal transduction pathway and mitogen-activated protein kinases (MAPK) cascade, type-2C protein phosphatase (PP2C) plays crucial roles in plant responses to abiotic stresses. However, the *PP2C* gene family’s responses to abiotic stress in litchi (*Litchi chinensis* Sonn.) have not been systematically studied. In this study, we predicted the 68 *PP2C* (designated *LcPP2C*) genes randomly distributed across fourteen chromosomes in the litchi genome. Phylogenetic tree analysis among litchi, Arabidopsis (*Arabidopsis thaliana*), and rice (*Oryza sativa*) revealed that the phylogenetic tree was divided into thirteen groups (A, B, C, D, E, F1, F2, G, H, I, J, K, and L). Closely linked *LcPP2C* genes within the same group exhibited various similarities in gene structures and motif compositions. Collinearity analysis demonstrated that segmental duplication (SD) events were the main dramatically increasing numbers in the *LcPP2C* gene family members. Cis-acting element analysis revealed that the 68 *LcPP2C* genes contained hormone and stress response elements with varying quantities, implying their potential in litchi stress resistance. Expression analysis showed that all the *LcPP2C* genes exhibited varying expression levels across nine different litchi tissues, more than 50% of genes within each group displayed similar tissue-specific expression patterns. The expression intensity, duration and regulation direction (up- or down-regulation) of the *LcPP2C* genes were varied under different abiotic stresses (cold, heat, and drought). The physiological and biochemical tests indicated that eight activation indexes (peroxidase (POD), catalase (CAT), superoxide dismutase (SOD), malondialdehyde (MDA), proline (PRO), soluble protein (SP), hydrogen peroxide (H_2_O_2_), and soluble sugar (SS)) increase at different level. Additionally, we analyzed physicochemical properties, subcellular locations, and secondary structures of the *LcPP2C* family members. Notably, the extensive connectivity of *LcPP2C32*/*60*/*9*/*37* underscored their vital roles in orchestrating and regulating biomolecular networks. These results provide valuable information for the identification of the *LcPP2C* genes and ideas for the cultivation of its transgenic induction lines in litchi.

## 1 Introduction

Numerous reports have demonstrated that plants have evolved a series of interrelated regulatory mechanisms to perceive and respond to environmental stresses. Such response and adjustment occur through an array of signal pathways (Cao et al., 2016; Gong et al., 2020; Zhang et al., 2022a). Reversible protein phosphorylation, mediated by protein kinases (PKs) and protein phosphatases (PPs), is one of the most vital post-translational modifications. It increases the structural complexity of protein and enhances their functional capabilities, thereby playing a pivotal role in stress regulation of plants (Bhaskara et al., 2019). Previous studies have shown the number of PKs is often higher than that of PPs in organisms. For instance, Arabidopsis (*Arabidopsis thaliana*) was found to encode approximately 1000 PKs (Smoly et al., 2017), but only about 150 PPs (Fuchs et al., 2013). Significantly, PKs usually receive more scholarly attention in signal transduction studies and have been established as positive regulatory factors responding to a variety of biotic and abiotic stresses (Hulsmans et al., 2016; Ahn et al., 2019; Dubrovinaa et al., 2015; Wu et al., 2015; Wang et al., 2016). In contrast, research on PPs is less extensive.

PPs are a type of enzyme molecule that catalyze the dephosphorylation reaction of phosphorylated proteins (Wei et al., 2014). They are classified into three major classes, namely, Ser/Thr phosphatases (STPs), protein Tyr phosphatases (PTPs), and dual-specificity phosphatases (DSPTPs) according to their substrate specificities (Shazadee et al., 2019). STPs are further categorized into phosphoprotein phosphatases (PPPs) and phosphoprotein metalloproteinases (PPMs) depending on different dependencies on metal ions, different amino acid sequences, and sensitivity to specific inhibitors like okadaic acid and cyclosporine-A (Haider et al., 2019). Furthermore, PPPs include type 1 (PP1), type 4 (PP4), type 5 (PP5), type 6 (PP6), type 7 (PP7), type 2A (PP2A), and type 2B (PP2B), whereas PPMs mainly involve Mg^2+^- or Mn^2+^-dependent type 2C protein phosphatases (PP2Cs) and pyruvate dehydrogenase phosphatases (Wang et al., 2021). Within PPMs, PP2C is an evolutionarily conserved and widely present in archaea, bacteria, fungi, plants, and animals in the form of monomeric enzymes (Yang et al., 2018). Interestingly, plants have a greater number of PP2Cs compared to other organisms (Singh et al., 2015). Additionally, most plant PP2Cs have conserved C-terminal domains with catalytic function, while N-terminus contain non-conservative region of varying lengths, which may be one of the reasons for their functional differentiation (Schweighofer et al., 2004).

PP2Cs catalyze the dephosphorylation of substrate protein molecules to regulate various signal transduction pathways and contribute to a wide range of physiological and biochemical processes in plants as an important class of PPs (Cao et al., 2016). Among various plant species, Arabidopsis serves as a particularly representative model for PP2C research, given its well-characterized genome and well-defined roles of its *PP2C* family members in numerous biological processes. In Arabidopsis, a total of 80 *AtPP2C* genes have been predicted and divided into twelve groups (A-L), excluding seven unclassified members (Fuchs et al., 2013). Within this classification, group A genes act as key negative regulators in abscisic acid (ABA) signaling pathways, interacting with ABA receptor proteins (pyrabactin resistance (PYR)/pyr1-like (PYL)/regulatory component of aba receptor (RCAR)) and sucrose nonfermenting 1 (SNF1) -related protein kinase 2s to modulate processes such as seed germination and dormancy, seedling root growth, stomatal movement, and fruit development (Park et al., 2009; Finkelstein, 2013; He et al., 2021); APC1 and AP2C3 of group B can regulate stomatal development and change several hormones level by negatively regulating mitogen-activated protein kinases (MAPKs) signaling in plants (Schweighofer et al., 2007; Umbrasaite et al., 2010); poltergeist (POL) and poltergeist-like1-5 (PLL1-5) of group C regulate the development of nutritive tissues and floral meristems as negative regulatory factors of clavata (CLV1) signaling pathways (Song et al., 2006); PP2C.D of group D inhibits cell expansion by physically interacting with small auxin up RNA (SAUR) proteins and PM H^+^-ATPase, thereby affecting plant growth and development (Wong et al., 2021); *PP2C6-6* of group E interacts with histone acetyl transferase (AtGCN5) to modulate stomatal signaling (Servet et al., 2008); WIN2 of group F interacts with the bacterial effector HopW1-1 to induce stress response (Wang et al., 2021); and the unclassified *PP2C* genes interact with receptor-like kinases (RLKs) to regulate hormonal signaling and effector-triggered immunity (ETI) (Haider et al., 2019).

Likewise, there have been an increasing number of research projects to study the role of PP2Cs in plant resistance to abiotic stresses by participating in the regulation of ABA signaling pathways (Yu et al., 2019). In moss (*Bryophyta*), PpABI1A and PpABI1B directly participated in ABA reactions to induce vegetative desiccation tolerance (Komatsu et al., 2013). In tobacco (*Nicotiana tabacum*), overexpression of maize (*Zea mays*) *ZmPP2C2* had germination percentage, germination rate, and antioxidant enzyme activity, thereupon then enhanced tolerance to cold stress, which indicates that *ZmPP2C2* may be a positive regulatory factor of plant resistance to low temperature stress (Hu et al., 2010). In Arabidopsis, highly ABA-induced *PP2C* gene 1 (HAI1) and ATK1 interacting protein phosphatase 1 (AIP1) of group A could regulate ABA sensitivity to cope with drought stress by interacting with SAUR32 (He et al., 2021); *FGT2* gene of group D interacted with phospholipase D α2 (PLDα2) to participate in the regulation of memory under high temperature stress (Castellanos et al., 2020); PP2CG1 of group G interacted with open stomata 1 (OST1) to negatively regulate the cold tolerance (Lv et al., 2021). Another study reported that *OsSIPP2C1* negatively regulated by ABL1 is involved in abiotic stresses (salt and drought) and panicle development in rice (*Oryza sativa*) (Li et al., 2013) Taken together, the studies mentioned above have demonstrated the diverse roles of *PP2C* genes in plants development and environmental stresses. Hence, it is necessary to delve into the characteristics and functions of *PP2C* gene family members, which will lay the foundation for elucidating the underlying molecular mechanisms of *PP2C* genes in stress signal transduction.

Litchi (*Litchi chinensis* Sonn.) belonging to family Sapindaceae, is a subtropical and tropical evergreen fruit tree. It has excellent flavor, rich nutrition, and far-reaching medical, is one of the most important and economically valuable fruit crops in the world (Yang et al., 2024a). With the completion of the litchi genome sequencing (Hu et al., 2022), many genes related to flowering or stress-tolerance have been thoroughly studied in litchi (Yang et al., 2024a; Ding et al., 2015; Xiao et al., 2018; Hu, 2018; Guan et al., 2021), but researches on the *PP2C* gene family have not been carried out yet. In the present study, the genome-wide identification of a full set of the *PP2C* gene family in litchi was conducted, summarized, and named. We then systematically analyzed physicochemical properties, genetic phylogeny, gene structures, collinearity relationships, cis-acting elements, and expression patterns in different tissues. Finally, we compared the expression characteristics of the *PP2C* genes and physiological and biochemical indexes in litchi under various abiotic stresses (cold, heat, and drought) were analyzed and compared. The findings will provide a standpoint for the identification of the *LcPP2C* gene family and set a foundation for further exploring response mechanism to abiotic stress of the various *LcPP2C* genes, which can provide a reference for developing abiotic resistant litchi cultivars.

## 2 Materials and methods

### 2.1 Plant materials and stress treatments

An annual early-maturing variety of litchi, ‘Feizixiao’ (wide planting areas, high yield, and strong adaptability in China) (Yang et al., 2024b), was obtained from the Institute of Fruit Tree Research, Guangdong Academy of Agricultural Sciences, situated at coordinates 113^°^22′41.200′′ E, 23^°^9′32.418′′ N, and an elevation of 1210 m, in Guangzhou City, Guangdong province, China. The seedlings were cultured in circular plastic flowerpots (size 26.5 cm × 17.5 cm × 21 cm, one plant per pot) containing sandy red soil, peat soil, and coconut bran silk (3:1:1 ratio, with pH values between 5.5 and 6.5) and routinely managed in a greenhouse. One-year-old seedlings with twenty-five leaves were selected for the stress treatment experiments in April 2023. To investigate the responses of litchi seedlings to cold stress, heat stress, and drought stress (fifteen seedlings per stress), the stresses included 4.0 ± 1.0°C, 38.0 ± 0.5°C, and 20% (*w*/*v*) PEG6000, based on the growth conditions of litchi in complex and variable environments (Yang et al., 2024). Specifically, fifteen seedlings were subjected to cold stress by being placed in an incubator set at 4.0 ± 1.0°C under a photoperiod of 16 h light/8 h dark; another 15 seedlings were exposed to heat stress in an incubator maintained at 38.0 ± 0.5°C under the same photoperiod; while the remaining 15 seedlings were irrigated with 20% (*w*/*v*) PEG6000 solution in a greenhouse setting. For samples used for RNA extraction, upper young leaves were harvested at 0, 3, 6, 12, and 24 h after stresses. Three biological replicates were prepared, whereas each of them gathered samples from three individual seedlings. All the harvested leaves were instantly frozen in liquid nitrogen and placed at -80°C for subsequent experiments.

### 2.2 Retrieval and domain identification of the *LcPP2C* genes

To identify all members of the PP2C family in litchi, a Hidden Markov Model (HMM) profile of the PP2C domain (PF00481) was first downloaded from the protein family (Pfam) database (https://www.ebi.ac.uk/). Secondly, a local HMMER 3.0 search was run with the expected value (E-value) of 1 × 10^-3^ utilizing the HMM profile as the query in the litchi genome database (Hu et al., 2022). Thirdly, these presumed LcPP2C sequences were submitted to a Batch CD-search (https://www.ncbi.nlm.nih.gov/cdd/?term=), an InterPro search (https://www.ebi.ac.uk/interpro/search/sequence/), and a SMART search (http://smart.embl-heidelberg.de/) to identify their conserved domains.

### 2.3 Physicochemical properties and subcellular locations of the LcPP2C proteins

The LcPP2C protein sequences were submitted to the ExPASy ProtParam tool (https://web.expasy.org/protparam/) to predicted their basic physicochemical properties, including coding sequence (CDS) size, protein size, molecular mass, theoretical isoelectric point (pI), instability index, and grand average of hydropathicity (GRAVY). In addition, the Protein Subcellular Localization Prediction Tool (PSORT) (https://www.genscript.com/psort.html) was employed to evaluate the subcellular locations of the LcPP2C proteins.

### 2.4 Phylogenetic analysis, structure information, and motif composition of the *LcPP2C* genes

The formation of an unrooted Neighbor-Joining (NJ) tree for PP2C protein sequences in litchi, Arabidopsis (**Supplementary Table S1**), and rice (**Supplementary Table S2**), was constructed using the MEGA 7.0 software with 1 000 bootstrap replications. The GSDS 2.0 server (http://gsds.gao-lab.org/) was utilized to perform a structural map of the *LcPP2C* genes. Subsequently, the conserved motifs of the *LcPP2C* genes were discovered using the MEME sever (https://meme-suite.org/meme/tools/meme) with the maximum motif number of 14 and were generated using a Visualize MEME/MAST Motif Pattern plugin from the TBtools-II v2.096 (Chen et al., 2023).

### 2.5 Chromosomal distribution and colinear analysis of the *LcPP2C* genes

The *LcPP2C* gene family members were localized to the litchi chromosomes using a Gene Location Visualize from GTFIGFF plugin from the TBtools-II v2.096 based on the litchi genome annotation (Hu et al., 2022). The homologous gene pairs and syntenic relationships among *PP2C* family members in litchi compared to itself, as well as between litchi and other species, including Arabidopsis, longan (*Dimocarpus longan*), apple (*Malus domestica*), rice, banana (*Musa cavendish*), and pineapple (*Ananas comosus*) were obtained using a One Step MCScanX plugin from the TBtools-II v2.096 with default parameters, an Advanced Circos plugin and a Dual Systeny Plot plugin from the TBtools-II v2.096 were then used to construct the collinear maps of litchi with itself and litchi with other species mentioned above, respectively. And besides, a Simple Ka/Ks Calculator (NG) plugin from the TBtools-II v2.096 was used to calculate the rates of nonsynonymous substitution (Ka) and synonymous substitution (Ks). Ka/Ks rate > 1 indicates positive evolution, Ka/Ks rate = 1 indicates neutral evolution, and Ka/Ks rate < 1 indicates negative evolution.

### 2.6 Protein-protein interaction network and cis-acting elements of the *LcPP2C* genes

The interaction network of the LcPP2C proteins was visualized using a STRING search (https://cn.string-db.org/) with Arabidopsis species and 0.40 confidence. Then, the cis-acting elements of the *LcPP2C* genes were predicted using a PlantCARE search (http://bioinformatics.psb.ugent.be/webtools/plantcare/html/) and were graphed using a HeatMap plugin from the TBtools-II v2.096.

### 2.7 Tissue-specific expression and qRT-PCR analysis of the *LcPP2C* genes

The expression profiles of the *LcPP2C* genes in different tissues, including roots, leaves, male flowers, female flowers, ovaries, carpopodiums, pericarp, fruitlets, aril, and seeds, were obtained from the litchi genome database (Hu et al., 2022) and were visualized using a HeatMap plugin with the FPKM (Fragments Per Kilobase of transcript per Million mapped reads) normalizing. Moreover, extraction and reverse transcription of total RNA were performed using the Plant Total RNA kit (SIMGEN, Hangzhou, China) and cDNA First Strand Synthesis kit (SIMGEN, Hangzhou, China). Furthermore, the 2 × SYBR Green PCR Mix kit (SIMGEN, Hangzhou, China) and QuantStudio™ 3 Real-Time PCR system (Thermo Fisher Scientific, Waltham, America) were manipulated to conduct the quantitative real-time PCR (qRT-PCR) analysis of the *LcPP2C* genes. The final data for each *LcPP2C* genes was calculated as an average of triplicate reactions using the 2^−ΔΔCt^ method. The relative expressions of the *LcPP2C* genes were significantly analyzed using the DPS 9.01 with *p* < 0.05 and were visualized using the SigmaPlot 14.0. The *Actin* gene (Wei et al., 2011) was chosen as the internal reference gene, and the list of primers used in this study is provided in **Supplementary Table S3**.

### 2.8 Physiological and biochemical characteristics of litchi seedlings under abiotic stresses

The activities of peroxidase (POD), catalase (CAT), and superoxide dismutase (SOD), and the contents of malondialdehyde (MDA), proline (PRO), soluble protein (SP), hydrogen peroxide (H_2_O_2_), and soluble sugar (SS), were determined using the respective POD, CAT, SOD, MDA, PRO, SP, H_2_O_2_, and SS test kits (BOXBIO, Beijing, China). The Spark^®^ 20M multimode microplate reader (Technical Analysis Equipment, Salzburg, Austria) was then used to measure the absorbance of different wavelengths and calculate enzyme activities or contents. The physiological and biochemical responses of litchi seedlings were significantly analyzed using the DPS 9.01 with *p* < 0.05 and were generated using the SigmaPlot 14.0.

## 3 Results

### 3.1 Identification and physicochemical property analysis of the *LcPP2C* genes

A total of 68 putative *PP2C* genes were found from the litchi genome depending on the HMMER search and conservative domain confirmation, arranged as *LcPP2C1* to *LcPP2C68* based on their locations on fourteen chromosomes (starting from the first on chromosome 1 labelled *LcPP2C1*). The basic characteristics of these 68 genes were summarized as shown in **Supplementary Table S4**. Specifically, the coding sequence (CDS) sizes of the 68 *LcPP2C* genes varied from 735 bp (*LcPP2C64*) to 3234 bp (*LcPP2C19*), with large variations in protein lengths from 244 aa (*LcPP2C64*) to 1077 aa (*LcPP2C19*) and molecular masses from 26.87 kDa (*LcPP2C64*) to 121.17 kDa (*LcPP2C24*). The theoretical pI values fluctuated between 4.60 (*LcPP2C32*) and 9.61 (*LcPP2C9*), with the majority (the 52 *LcPP2C* genes) of theoretical pI values < 7.00, signifying that this gene family was biased towards acidic proteins. The instability indexes changed significantly between 30.08 (*LcPP2C47*) and 64.76 (*LcPP2C40*), with the majority (the 47 *LcPP2C* genes) of instability indexes > 40.00, suggesting that this gene family was mainly composed of unstable proteins. The GRAVY values in the 66 *LcPP2C* genes (except *LcPP2C60*/*63*) were less than zero, indicating that these genes were hydrophilic. Additionally, the subcellular locations were very diverse, with the 28 LcPP2C proteins being assigned to chloroplast, 18 as nucleus, 16 as cytoplasm, 4 as mitochondrion, 1 as vacuole, and 1 as plasma membrane.

### 3.2 Phylogenetic relation and classification of the *LcPP2C* genes

**Figure 1:**
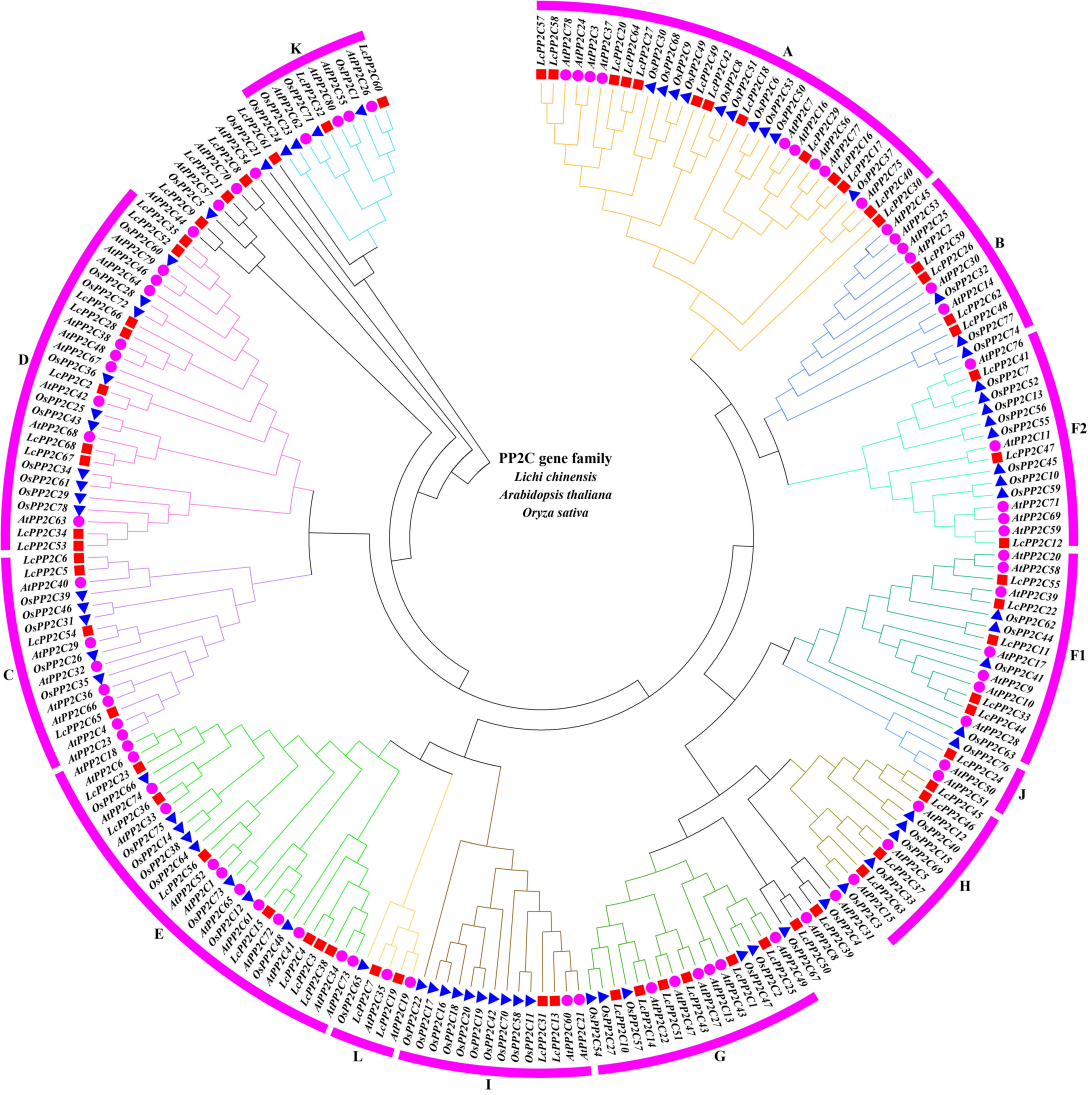
Phylogenetic tree of *PP2C* genes from litchi, Arabidopsis, and rice. Red squares represent the *LcPP2C* genes, purple circles represent the *AtPP2C* genes, and blue triangle represent the *OsPP2C* genes. **(A, B, C, D, E, F1, F2, G, H, I, J, K)**, and **(L)** represent different groups, and these groups are displayed in different colors.

To probe into the genetic phylogeny and the group classifications of the *LcPP2C* gene family, a phylogenetic tree was constructed by aligning the amino acid sequences of the 68 putative *LcPP2C* genes, the 80 reported *AtPP2C* genes, and the 78 reported *OsPP2C* genes (Xue et al., 2008) based on the classifications of two model plants (Arabidopsis and rice). The **Figure 1** displayed that the 68 *LcPP2C* genes were classified into thirteen groups (A, B, C, D, E, F1, F2, G, H, I, J, K, and L), excluding 6 unclassified genes. Among them, group A contained the most *LcPP2C* genes (13), followed by D (9), E (7), G (6), F1 (5), B (4), C (4), H (4), F2 (3), I (2), K (2), L (2), and J (1). As a whole, there was no genetic divergence between the *LcPP2C* family, *AtPP2C* family, and *OsPP2C* family, manifesting that *PP2C* genes are relatively conserved in genetic evolution.

### 3.3 Evolution, structures, and conserved motifs of the *LcPP2C* genes

**Figure 2:**
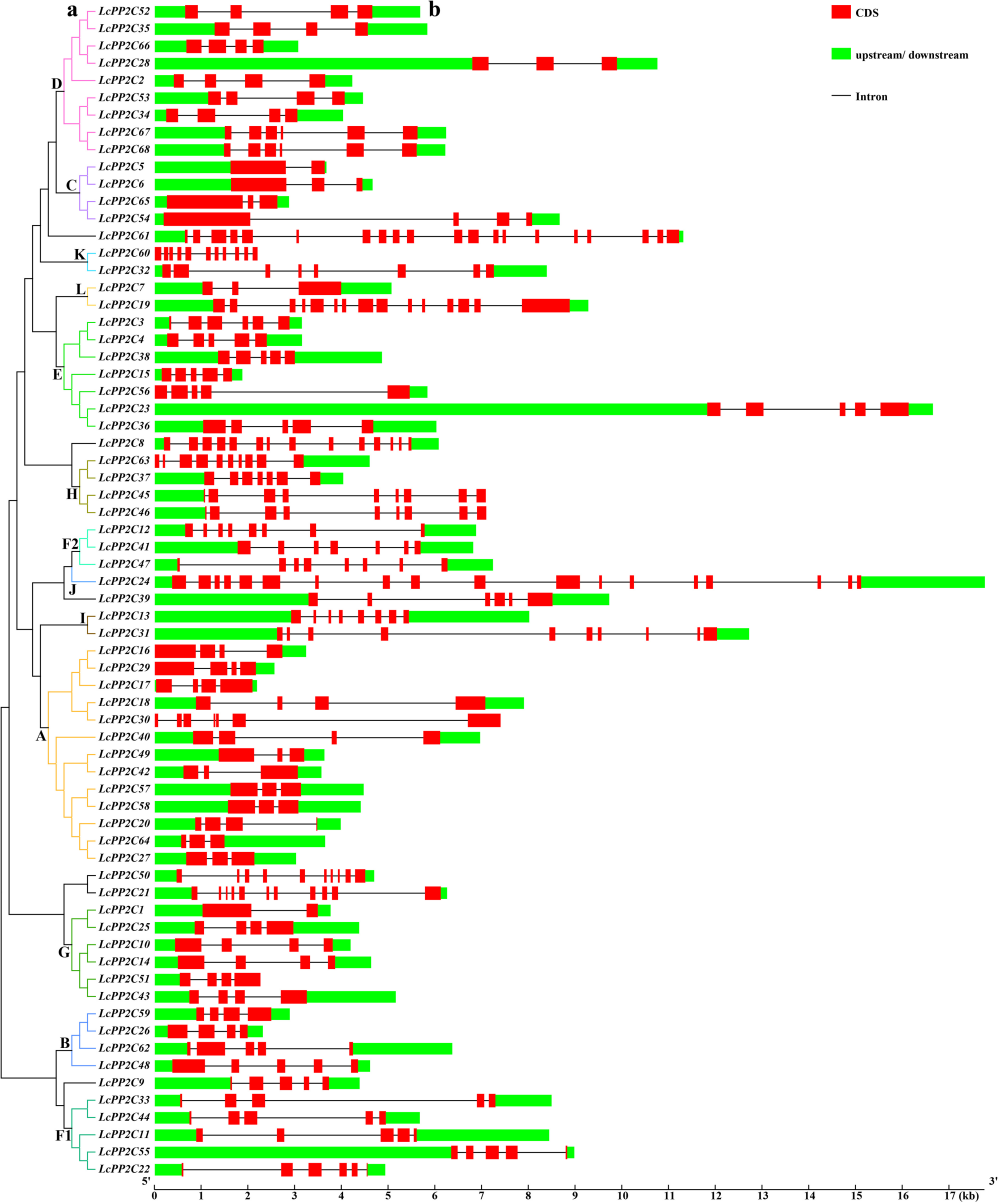
Phylogenetic relations and gene structures of the *LcPP2C* genes. **(a)** A phylogenetic tree was constructed using the full-length sequences of the LcPP2C proteins. **(b)** Exon–intron structures of the *LcPP2C* genes. Red boxes, black lines, and green boxes indicate exons, introns, and upstream or downstream, respectively.

To comprehend the structural composition of the *LcPP2C* gene family members, a phylogenetic tree was established based on the LcPP2C protein sequences, closely followed by gene structure information (**Figure 2**). The results of phylogenetic tree were broken out into thirteen groups (**Figure 2A**), which were consistent with the results of unrooted phylogeny (**Figure 1**). Comparison of the number and location for exons and introns illustrated that the 68 *LcPP2C* genes had relatively variable structure characteristics (**Figure 2B**). More generally, the *LcPP2C* family members contained exons ranging from two to twenty, and approximately the 64.71% (44) *LcPP2C* genes contained the numbers of exons spanned from three to five. Among these, *LcPP2C61* had the greatest number of introns for nineteen, while *LcPP2C1*/*5* had the smallest number of introns for one. Notably, more than 29% (20) among the *LcPP2C* genes all contained three introns, conjecturing that the structures of most their genes were conservative. Additionally, the *LcPP2C* genes that were clustered together generally contained similar structures; for example, all the members of group F1 contained five exons and four introns. Nevertheless, *LcPP2C3* was quite different from the other genes in group E, connoting that a different evolutionary type of this gene had undergone.

**Figure 3:**
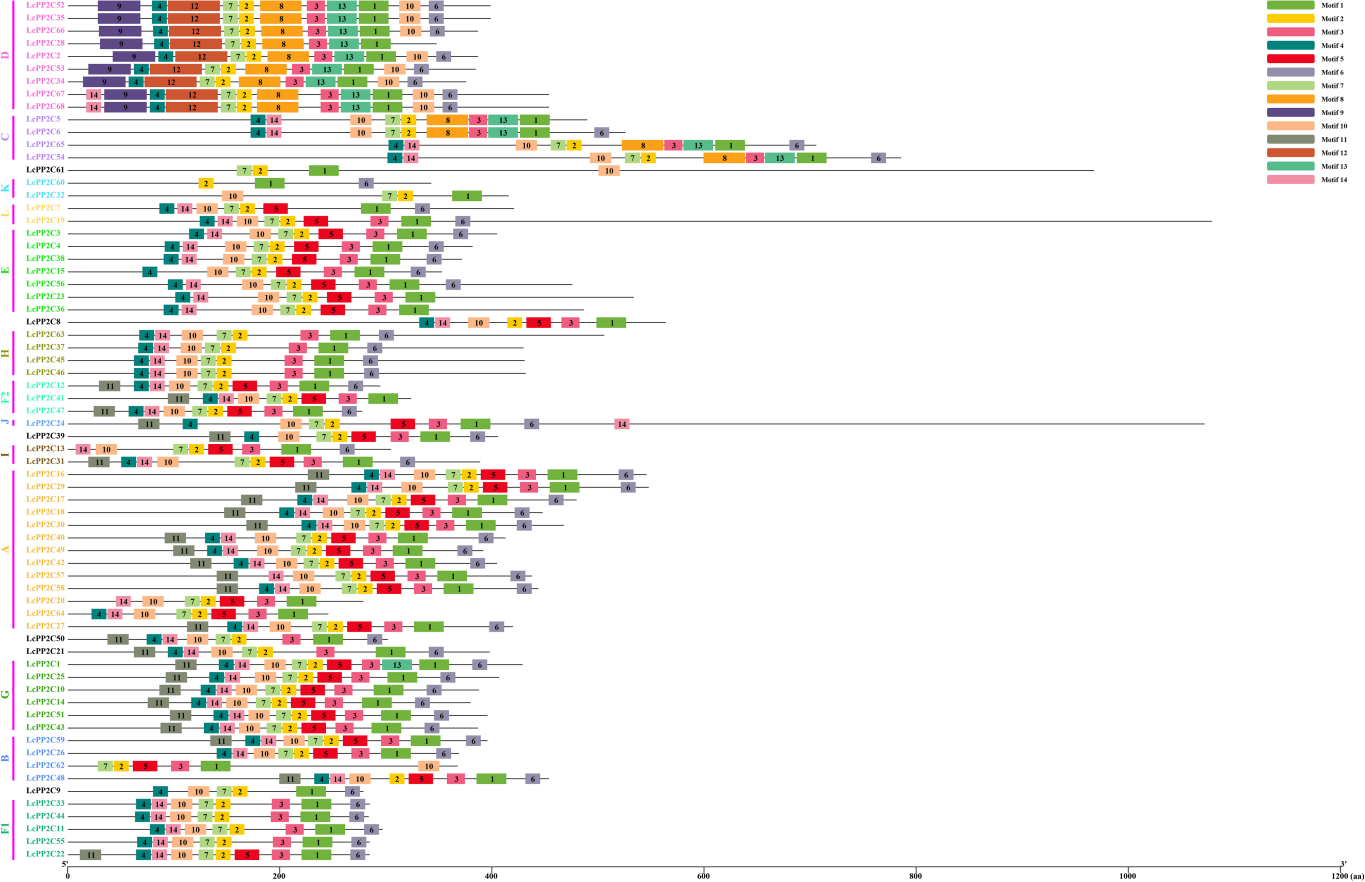
Motif pattern of the LcPP2C proteins. Fourteen putative motifs are indicated in different colored boxes, and black lines indicate amino acid length.

To further comprehend the diversity of changes in the *LcPP2C* gene family during evolution, a motif pattern map was depicted using the LcPP2C protein sequences. The results illustrated that fourteen conserved motifs were identified (**Figure 3**), whose detailed sequence information are provided in **Supplementary Table S5**. Obviously, Motif 1/2/3/4/5/6/7/8/9/10/11/12/13/14 were not simultaneously distributed among any of the *LcPP2C* genes, Motif 9/12 were unique to the members of group D, Motif 8 only existed in C and D members, and all the *LcPP2C* family members had Motif 1/2. Moreover, the *LcPP2C* members gathered typically had similar motif arrangements; for example, there were similar quantity, type, and spatial distribution among the motifs of all H members. However, *LcPP2C41* did not contain Motif 6 compared to other F2 members, conjecturing that *LcPP2C41* has lost bases during the tandem duplication (TD) process.

### 3.4 Chromosomal distribution and duplication events of the *LcPP2C* genes

**Figure 4:**
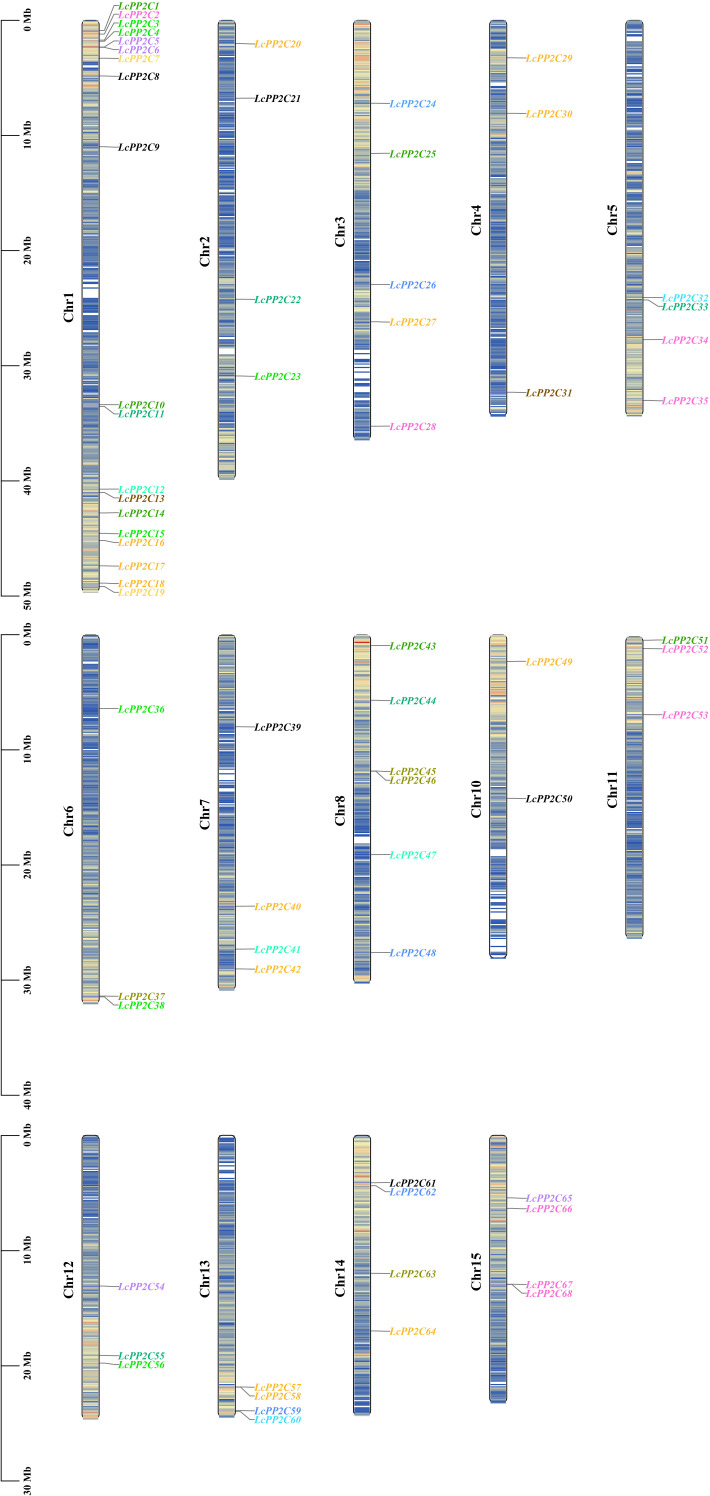
Chromosomal location of the *LcPP2C* genes. The colored rectangular bars represent the chromosomes of litchi. The chromosome numbers are displayed on the left side, the gene names are displayed on the right side, and the 0-50 Mb scale represents chromosome length. The gene names with different colors represent different groups.

To understand the distribution characteristics of the *LcPP2C* family members on chromosomes, a chromosomal positional map was constructed on the basis of the litchi genome annotation. The results indicated that the 68 *LcPP2C* genes were unevenly distributed across fourteen chromosomes (**Figure 4**; **Supplementary Table S4**). Among them, Chr1 contained 19 genes, Chr2 contained 4, Chr3 contained 5, Chr4 contained 3, Chr5 contained 4, Chr6 contained 3, Chr7 contained 4, Chr8 contained 6, Chr10 contained 2, Chr11 contained 3, Chr12 contained 3, Chr13 contained 4, Chr14 contained 4, and Chr15 contained 4. Clearly, there was no positive correlation between chromosome length and the number of genes contained on them. Unexpectedly, the different *LcPP2C* genes contained on the same chromosome were usually categorized as different groups in the evolutionary relationships within litchi, implying that the different *LcPP2C* genes contained on a chromosome may exert different functions.

**Figure 5:**
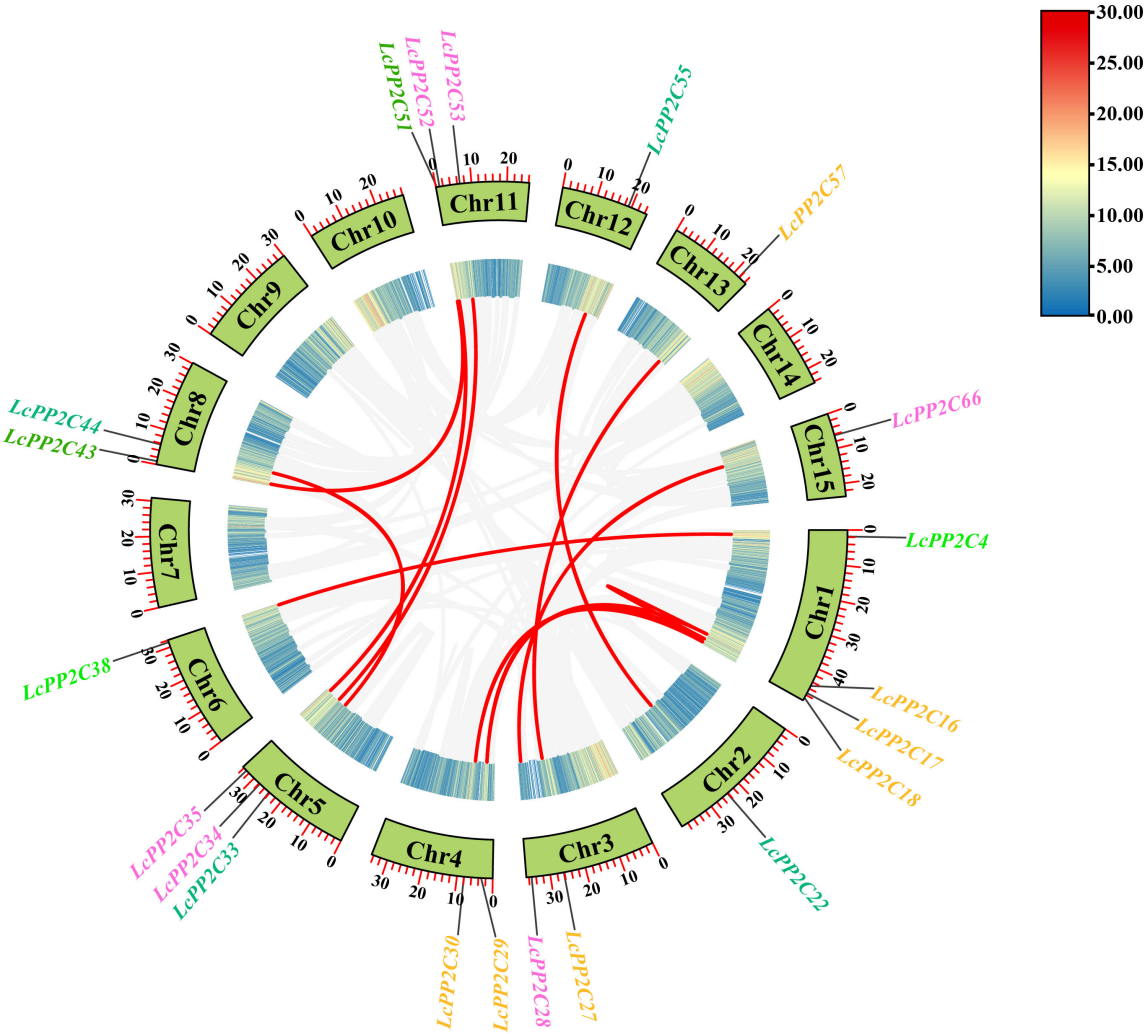
Chromosomal distribution and gene duplication relationships of the *LcPP2C* genes. The gray lines represent the collinear regions in the litchi genome, and the red lines represent the duplicated *LcPP2C* gene pairs. The Chromosomal number is shown inside each chromosome, and the genes with different colors represent different groups.

To investigate the expanded form of the *LcPP2C* gene family, the duplication relationships among the *LcPP2C* genes were identified. According to the chromosomal location information (**Supplementary Table S4**), no a pair of TD event was observed while twelve pairs of segmental duplication (SD) events were detected in the duplicated *LcPP2C* genes (**Figure 5**; **Supplementary Table S6**). It proved that the main drivers for the *LcPP2*C genes amplification were SD events. Expectedly, all the *LcPP2C* genes that underwent SD events belonged to the same group, suggesting that these genes may have worked together to encode proteins and regulate related biological processes. Among these, five pairs of the *LcPP2C* genes (*LcPP2C16*/*17*, *LcPP2C16*/*29*, *LcPP2C17*/*29*, *LcPP2C18*/*30*, and *LcPP2C27*/*57*) belonged to group A, three pairs (*LcPP2C28*/*66*, *LcPP2C34*/*53*, and *LcPP2C35*/*52*) belonged to D, two pairs (*LcPP2C22*/*55* and *LcPP2C33*/*44*) belonged to F1, and the remaining two pairs (*LcPP2C4*/*38* and *LcPP2C43*/*51*) belonged to E and G, respectively. The proportion of non-synonymous (Ka) to synonymous (Ks) in the duplicated *LcPP2C* gene pairs were then computed. The final outcomes suggested that the Ka/Ks ratios were all less than one (0.0680654 to 0.3190254) (**Supplementary Table S7**), meaning that purifying selection played a key role during the *LcPP2C* genes replication.

**Figure 6:**
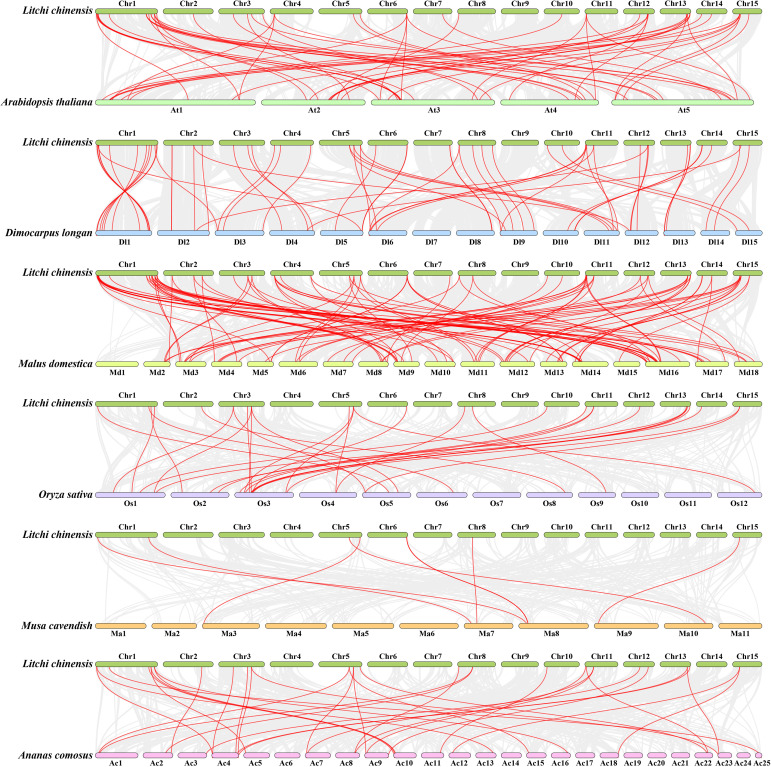
Collinear relationships of *PP2C* genes between litchi and six plants (Arabidopsis, longan, apple, rice, banana, and pineapple). The gray lines between litchi and other plants represent collinear blocks in wide regions of the genomes, while red lines represent the orthologous relationships of *PP2C* genes.

To further investigate the evolutionary relationships of *PP2C* genes between litchi and different species, six intergenomic collinear maps of litchi with three dicotyledons (Arabidopsis, longan, and apple) and three monocotyledons (rice, banana, and pineapple) were generated. The *LcPP2C* genes were found to have a closer collinear relationship with *PP2C* genes of these dicotyledons, with the strongest relationships in apple (one hundred and two pairs), followed by longan (sixty-one pairs), and lastly Arabidopsis (fifty-five). In contrast, the *LcPP2C* gene family shared only a few genes with PP2C family of these monocotyledons, including thirty-three pairs with pineapple, twenty-eight with rice, and eight with banana (**Figure 6**; **Supplementary Tables S8–13**). *LcPP2C3*/*7*/*8*/*10*/*13*/*20*/*24*/*28*/*32*/*39*/*41*/*44*/*46*/*58*/*64*/*68* did not have collinear *PP2C* genes with any of these six species, inferring that these *LcPP2* genes may have formed after plant differentiation.

### 3.5 Interaction network of the *LcPP2C* genes

**Figure 7:**
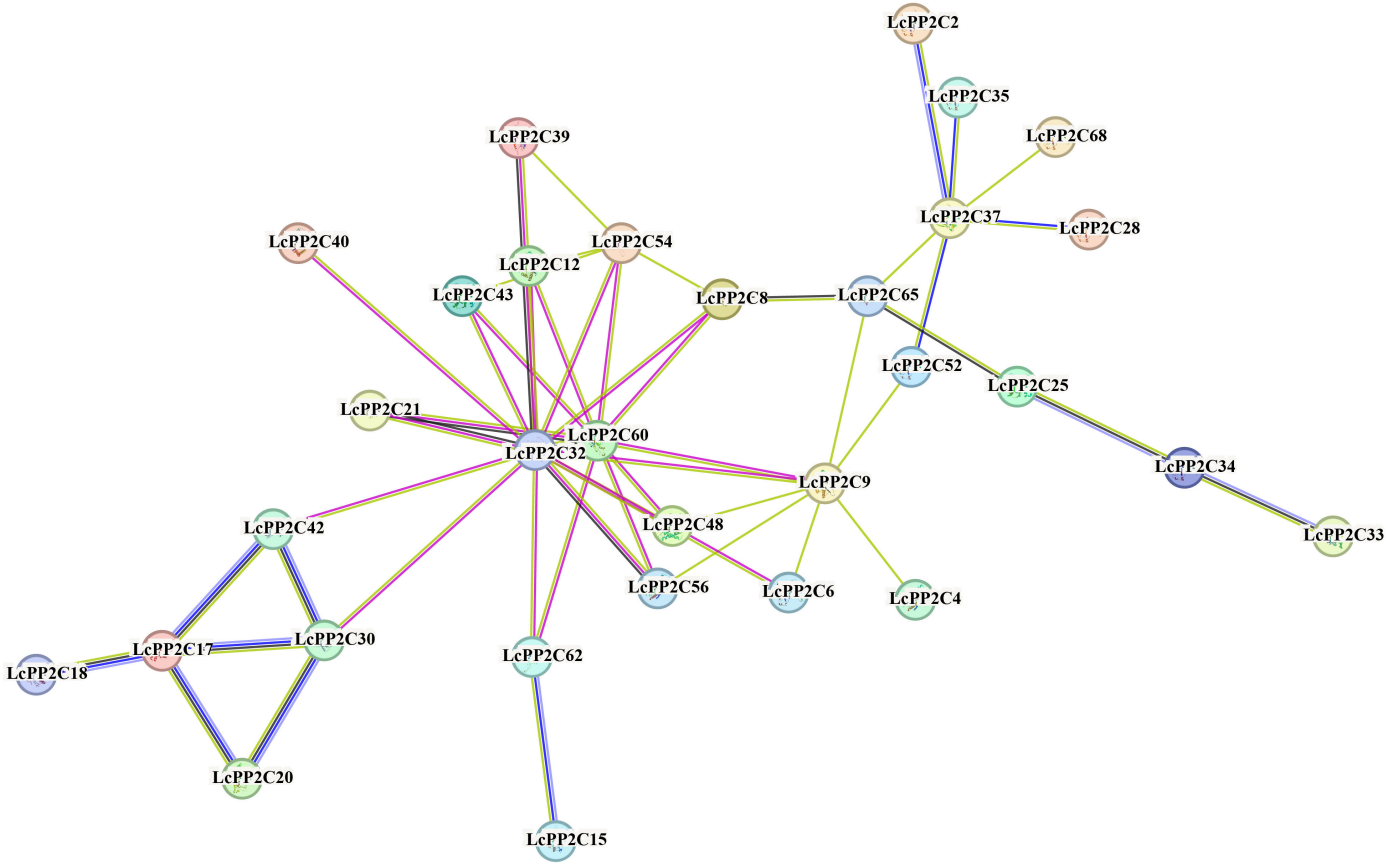
Protein-protein interaction network of the LcPP2C proteins. Nodes connected by light blue and purple lines represent known interacting proteins, the nodes connected by green, red and dark blue lines represent proteins that may interact.

To elucidate the possible working mechanism of the *LcPP2C* family, a protein-protein interaction network was explored using the STRING program based on the Arabidopsis association model. The results revealed that the 31 LcPP2C proteins (LcPP2C2/4/6/8/9/12/15/17/18/20/21/25/28/30/32/33/34/35/37/39/40/42/43/48/52/54/56/60/62/65/68) had close interaction relation, and there were fifty-one interactive relationships between them (**Figure 7**). Among these, LcPP2C32/60/9/37 were more critical among the entire network, and interacted with the 13 (LcPP2C8/9/12/21/30/40/42/43/48/54/56/60/62), 10 (LcPP2C8/9/15/21/32/43/48/54/56/62), 8 (LcPP2C4/6/32/48/52/56/60/65), and 6 (LcPP2C2/28/35/52/65/68) proteins, respectively. It is notable that the remaining 37 LcPP2C proteins were not found to involve in the interactions under the known parameters, implying that these proteins may independently exert regulatory effects.

### 3.6 Promoter region analysis of the *LcPP2C* genes

**Figure 8:**
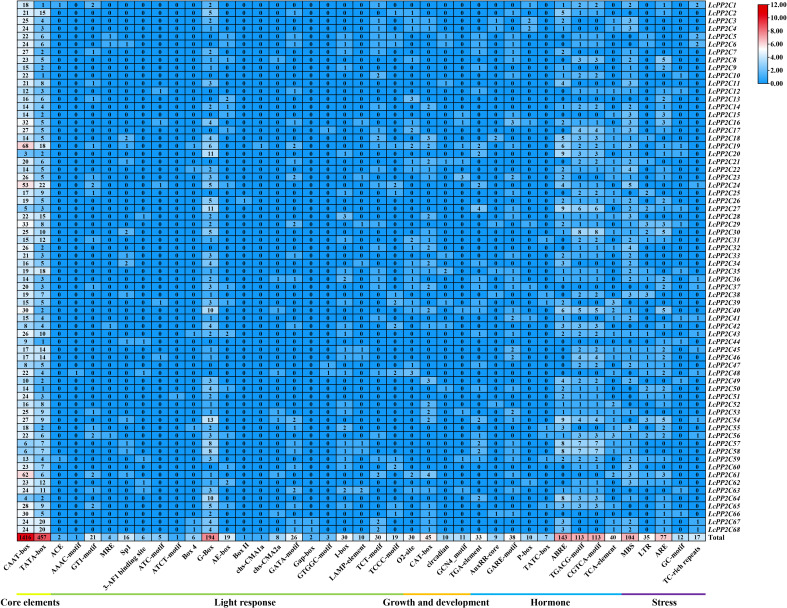
Cis-acting element analysis in the promoter regions of the *LcPP2C* genes. The number of different elements in the promoter regions of the *LcPP2C* genes, as indicated by different color intensities and numbers in the grid.

For a deep understanding of transcriptional regulation and potential functions for the *LcPP2C* genes, their cis-acting elements were predicted. It was found that 41 various classes of 3125 elements among the promoter regions of the *LcPP2C* genes were recognized (**Figure 8**; **Supplementary Table S14**). These elements then broke down into five main types, dealing firstly with 2 classes of 1873 basic cis-regulatory elements (CAAT-box and TATA-box), secondly with 21 classes of 405 light-related ones (ACE, AAAC-motif, GT1-motif, MRE, Sp1, 3-AF1 binding site, ATC-motif, ATCT-motif, Box 4, G-Box, AE-box, Box II, chs-CMA1a, chs-CMA2a, GATA-motif, Gap-box, GTGGC-motif, I-box, LAMP-element, TCT-motif, and TCCC-motif), thirdly 4 classes of 96 with growth-related ones (O2-site, CAT-box, circadian, and GCN4_motif), fourthly with 9 classes of 506 hormone-related ones (TGA-element, AuxRR-core, GARE-motif, P-box, TATC-box, ABRE, TGACG-motif, CGTCA-motif, and TCA-element), and fifthly with 5 classes of 245 stress-related ones (MBS, LTR, ARE, GC-motif, and TC-rich repeats). In fact, basic elements had the largest number (1873) and unevenly occurred in the 68 *LcPP2C* genes. Equally, there were 50, 355, 30, 45, 10, 11, 42, 55, 143, 226, 40, 104, 35, 77, 12, and 17 elements related to light responsiveness, part of a light response, zein metabolism regulation, meristem expression, circadian control, endosperm expression, auxin, gibberellin, abscisic acid, methyl jasmonate, salicylic acid, drought, low temperature, anaerobic induction, anoxic induction, and defense and stress, respectively. The results of promoter region analysis suggested that the *LcPP2C* family may participate in the responding of multi-factors, involving light, growth and development, hormones, and stresses, and these response elements may directly regulate the expression of the *LcPP2C* genes in the growth and development as well as various stresses.

### 3.7 Expression patterns of the *LcPP2C* genes in different tissues

**Figure 9:**
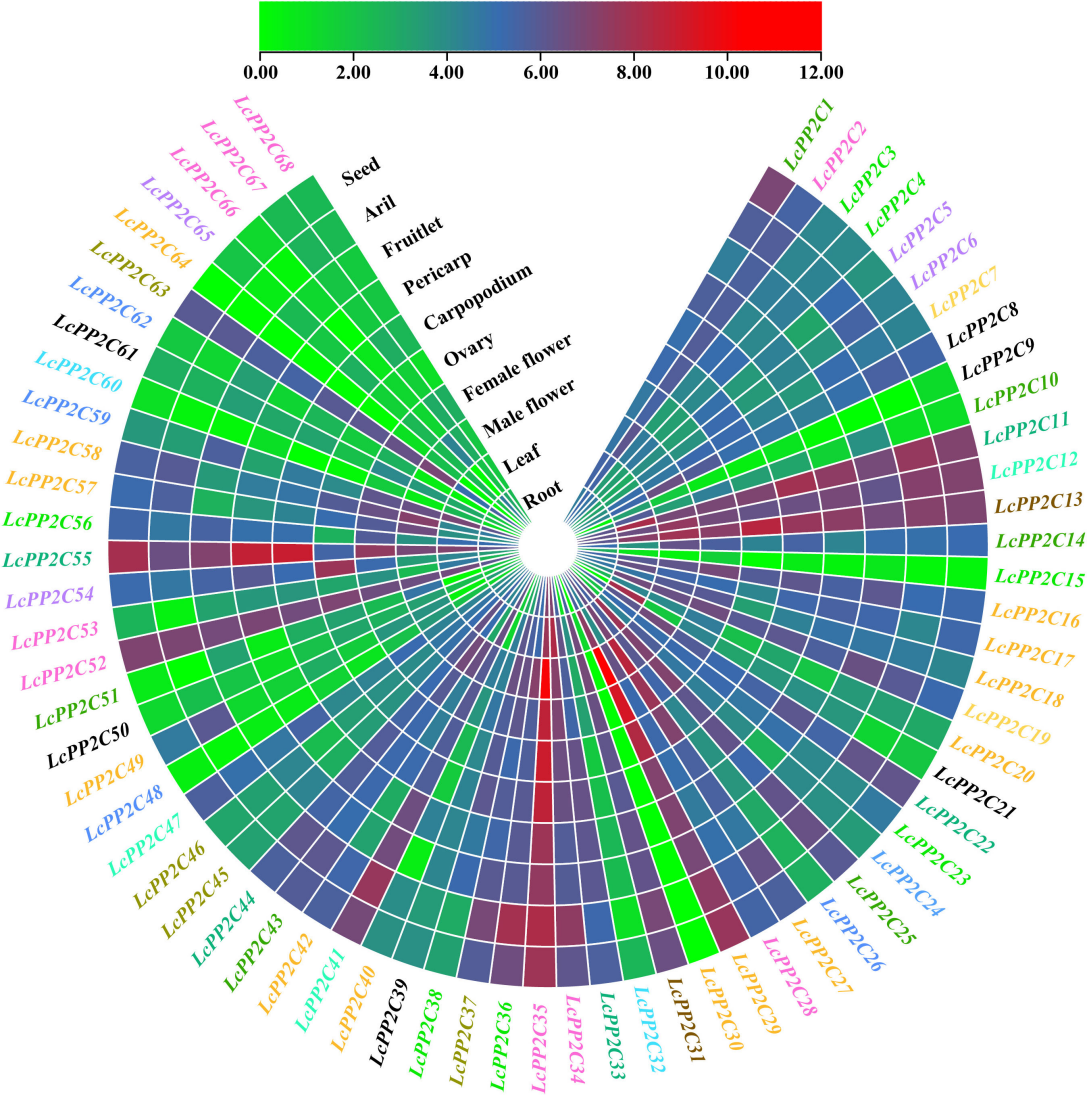
Expression patterns of the *LcPP2C* genes in ten tissues (roots, leaves, male flowers, female flowers, ovaries, carpopodiums, pericarp, fruitlets, aril, and seeds) for litchi. Red color represents induced expression and green represents repressed expression. The genes with different colors represent different groups.

To further establish the possible roles of the *LcPP2C* family members in growth and development of litchi, their expression levels in ten tissues (roots, leaves, male flowers, female flowers, ovaries, carpopodiums, pericarp, fruitlets, aril, and seeds) were counted. The results uncovered that the *LcPP2C* genes had distinct tissue-specific expression and expressed in at least one of these tissues (**Figure 9**; **Supplementary Table S15**). Specifically, the 68 *LcPP2C* members were split into three categories (A, B, and C) according to the number of tissues they showed. Namely, the category A genes exhibited in only one tissue while had no fluctuation in others, *LcPP2C30* was exclusively expressed in leaves; the B genes expressed in some (with more than one whereas less than ten) tissues, *LcPP2C64* showed in two of the ten tissues, *LcPP2C9*/*51*/*66* showed in eight, and *LcPP2C15*/*60* showed nine; the C genes exhibited in all ten tissues, the remaining 61 genes. Besides, the *LcPP2C* genes gathered roughly had similar expression patterns; for example, group B, C, F1, F2, H, I, J, and L genes emerged more or less expression levels in ten tissues, manifesting that these genes may take part in the growth and development of litchi extensively. Meanwhile, several group A genes (*LcPP2C30*/*64*) exhibited in a few individual tissues, denoting that these genes may only participate in the growth and development of one part for litchi. As a consequence, the reason for genetic functional diversity may be that the *LcPP2C* members showed distinct expression patterns.

### 3.8 Expression patterns of the *LcPP2C* genes in response to stress treatments

**Figure 10:**
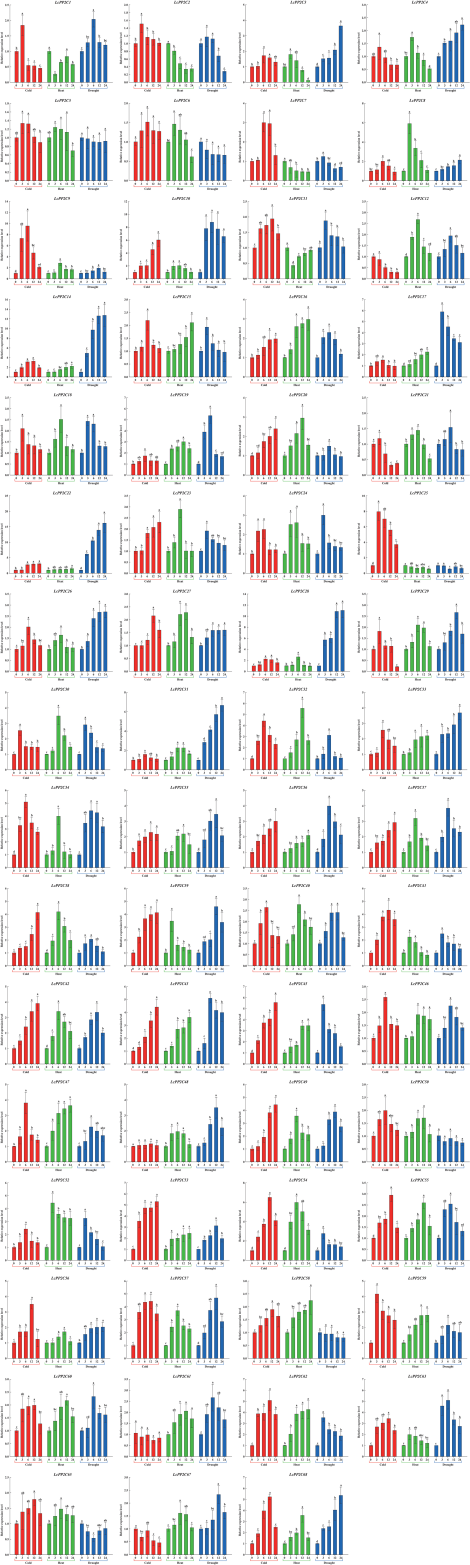
Relative expression of the *LcPP2C* genes under stress treatments in 0, 3, 6, 12, and 24 h. Red, green, and blue columns represent cold (4.0 ± 1.0°C), heat (38.0 ± 0.5°C), and drought (20% (*w/v*) PEG6000), respectively. Error bars represent the standard deviation of three replicates, and different lowercase letters represent significant differences (*p* < 0.05).

Summarizing the results of the cis-regulatory elements analysis (**Figure 8**; **Supplementary Table S14**) and the tissue-specific expression analysis (**Figure 9**; **Supplementary Table S15**), the 63 *LcPP2C* genes (*LcPP2C1*/*2*/*3*/*4*/*5*/*6*/*7*/*8*/*9*/*10*/*11*/*12*/*14*/*15*/*16*/*17*/*18*/*19*/*20*/*21*/*22*/*23*/*24*/*25*/*26*/*27*/*28*/*29*/*30*/*31*/*32*/*33*/*34*/*35*/*36*/*37*/*38*/*39*/*40*/*41*/*42*/*43*/*45*/*46*/*47*/*48*/*49*/*50*/*52*/*53*/*54*/*55*/*56*/*57*/*58*/*59*/*60*/*61*/*62*/*63*/*65*/*67*/*68*) were selected which exhibited in leaves and had multiple elements related to hormone (GARE-motif, P-box, TATC-box, ABRE, TGACG-motif, CGTCA-motif, and TCA-element) and stress (MBS, LTR, and TC-rich repeats) for detecting their relative expression in abiotic stresses (cold, heat, and drought). As a results, these 63 genes displayed differential expression after at least one stress treatment, but the treated time were not directly proportional to the degree of genetic responses (**Figure 10**).

In the cold treatment, the 60 *LcPP2C* genes (*LcPP2C1*/*2*/*3*/*4*/*5*/*6*/*7*/*8*/*9*/*10*/*11*/*14*/*15*/*16*/*17*/*18*/*19*/*20*/*21*/*22*/*23*/*24*/*25*/*26*/*27*/*28*/*29*/*30*/*31*/*32*/*33*/*34*/*35*/*36*/*37*/*38*/*39*/*40*/*41*/*42*/*43*/*45*/*46*/*47*/*48*/*49*/*50*/*52*/*53*/*54*/*55*/*56*/*57*/*58*/*59*/*60*/*62*/*63*/*65*/*68*) emerged with high expression compared to the untreated time (0 h) at a minimum of one treated time point, while 3 genes (*LcPP2C12*/*61*/*67*) had no significant induced differences. Among these, the 10 genes (*LcPP2C1*/*2*/*4*/*5*/*18*/*21*/*25*/*29*/*30*/*59*) reached their maximums at 3 h after treatment, the highest expression of the 36 genes (*LcPP2C3*/*6*/*7*/*8*/*9*/*11*/*14*/*15*/*17*/*19*/*24*/*26*/*27*/*28*/*31*/*32*/*33*/*34*/*35*/*40*/*41*/*46*/*47*/*48*/*50*/*52*/*54*/*55*/*56*/*57*/*58*/*60*/*62*/*63*/*65*/*68*) at 6 h or 12 h, and the 14 genes (*LcPP2C10*/*16*/*20*/*22*/*23*/*36*/*37*/*38*/*39*/*42*/*43*/*45*/*49*/*53*) were continuously up-regulated for 24 h. Likewise, during the heat treatment, the expression quantity of the 9 genes (*LcPP2C3*/*4*/*5*/*6*/*8*/*39*/*41*/*52*/*63*) were the highest at 3 h, the most remarkable expression of the 35 genes (*LcPP2C9*/*10*/*12*/*18*/*19*/*20*/*21*/*23*/*24*/*26*/*27*/*28*/*29*/*30*/*31*/*32*/*34*/*35*/*37*/*38*/*40*/*42*/*46*/*48*/*49*/*50*/*54*/*55*/*56*/*57*/*60*/*61*/*65*/*67*/*68*) could be seen at 6 h or 12 h, the 14 genes (*LcPP2C14*/*15*/*16*/*17*/*22*/*33*/*36*/*43*/*45*/*47*/*53*/*58*/*59*/*62*) showed over-expression that lasted to 24 h, and the 5 genes (*LcPP2C1*/*2*/*7*/*11*/*25*) weakly expressed compared to the 0 h at any heat time. Moreover, in the drought treatment, the expression of the 12 genes (*LcPP2C3*/*4*/*8*/*14*/*22*/*26*/*27*/*28*/*31*/*33*/*56*/*68*) started to increase after 3 h, and reached their maximums at 24 h. In contrast, the 6 genes (*LcPP2C5*/*6*/*25*/*50*/*58*/*65*) had a general downtrend between 0 h to 24 h. The highest expression levels of the 14 genes (*LcPP2C2*/*7*/*11*/*15*/*17*/*18*/*23*/*24*/*30*/*41*/*45*/*52*/*54*/*62*) were observed at 3 h, followed by the 20 genes (*LcPP2C1*/*10*/*12*/*16*/*19*/*20*/*21*/*32*/*34*/*36*/*37*/*38*/*43*/*46*/*47*/*55*/*59*/*60*/*61*/*63*) at 6 h, and the 11 genes (*LcPP2C9*/*29*/*35*/*39*/*40*/*42*/*48*/*49*/*53*/*57*/*67*) at 12 h. Taken overall, all these *LcPP2C* genes had different responses and involvement extents to experience cold, heat, and drought. It connoted that the *LcPP2C* gene family may exercise crucial roles in the regulation of abiotic stresses for litchi.

### 3.9 Physiological and biochemical responses of litchi seedlings to stress treatments

**Figure 11:**
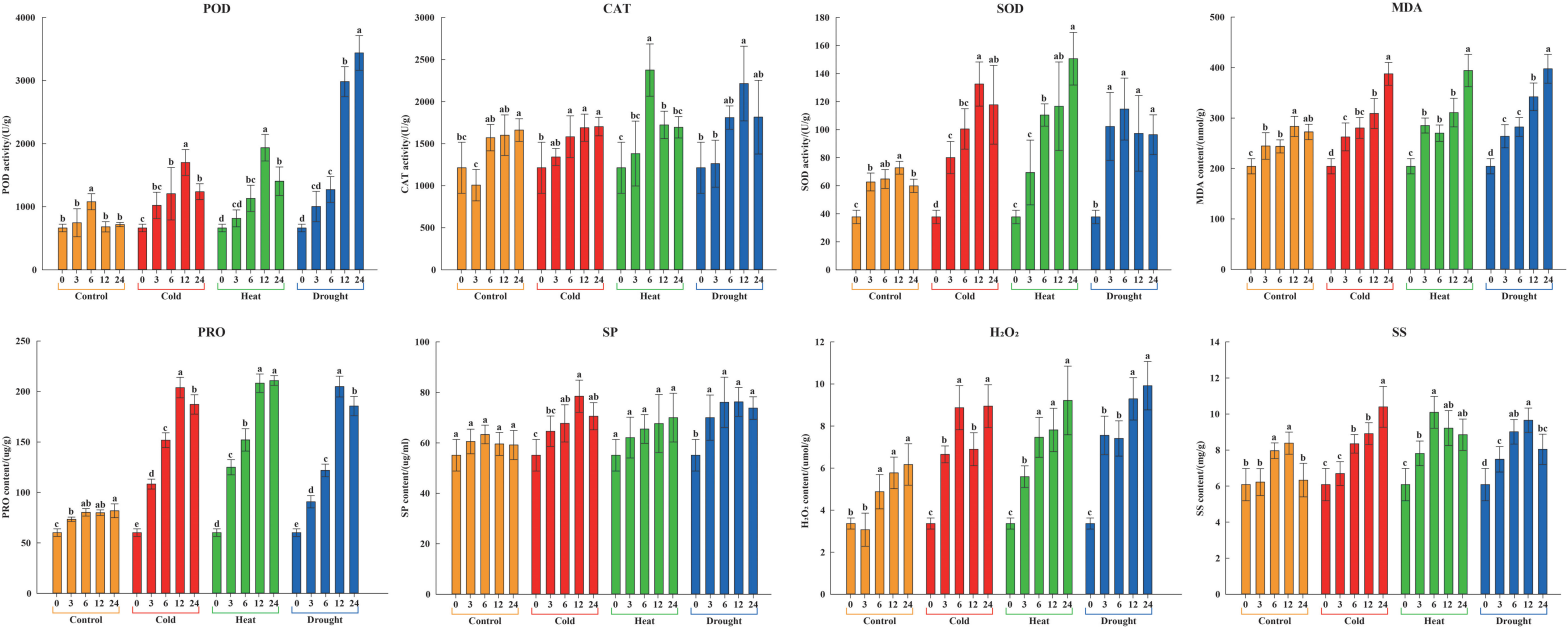
Physiological and biochemical characteristics of litchi seedlings under stress treatments in 0, 3, 6, 12, and 24 h. Yellow, red, green, and blue columns represent control, cold (4.0 ± 1.0°C), heat (38.0 ± 0.5°C), and drought (20% (*w/v*) PEG6000), respectively. Error bars denote the standard deviation of the mean. Different lowercase letters denote significant differences, whereas the same letters represent no significant differences at the 0.05 level.

To measure the resistant physiological changes of litchi under environmental stresses, their physiological and biochemical indices were detected. The results showed that the physiological responses of the stress-treated groups were faster and stronger than those of the untreated control at any given time (**Figure 11**).

After the cold treatment, POD, SOD, PRO, and SP rose to the highest levels at 12 h, beyond which they commenced a gradual decline. In contrast, CAT, MDA, H_2_O_2_, and SS continued to rise, attaining their peak concentrations at 24 h. After the heat treatment, CAT and SS accumulated rapidly in the first and had a peak at 6 h, closely followed by POD at 12 h, as well as SOD, MDA, PRO, SP, and H_2_O_2_ both at 24 h. After the drought treatment, there was a swift accumulation of SOD, which peaked at 6 h before steadily declining. This was succeeded by a surge in CAT, PRO, SP, and SS, all of which reached their acme at 12 h. Concurrently, the levels of POD, MDA, and H_2_O_2_ exhibited positive correlations with the overall duration of drought. In summary, the activities of POD, CAT, and SOD, and the contents of PRO, SP, and SS observed over treatment time were characterized by an initial ascent followed by a decline or them manifested as a sustained upward trajectory, while the contents of MDA and H_2_O_2_ showed an overall upward trend. Notably, these above indices were found to be significantly elevated compared to those in the control group at all measured time points. It proved that adverse environmental conditions could cause physiological and biochemical changes of litchi and induce high expression of the *LcPP2C* family members, suggesting that the *LcPP2C* gene family participated in the bioprocess of responding to abiotic stresses in litchi.

## 4 Discussion

PP2Cs have been proven to be one of the largest protein phosphatase families and get involved in a diverse range of physiological and biochemical processes (biosynthesis, hormone modulation, growth and development, and defense against stresses) in plants (Yu et al., 2019; Jung et al., 2020; Chen et al., 2022). Due to the rapid development of sequencing technologies, PP2C gene families have been reported in various species, including Arabidopsis (Xue et al., 2008), rice (Xue et al., 2008), grapes (*Vitis vinifera*) (He et al., 2018), strawberry (*Fragaria vesca*) (Haider et al., 2019), and apple (Wang et al., 2021). However, information and functions about the gene family that comprises the PP2C domain in litchi were still not studied. A high-quality genome from lichi (Hu et al., 2022) has provided a hard basis to conduct a comprehensive analysis and characterization of the *LcPP2C* gene family at the whole-genome level. Here, the 68 putative *LcPP2C* genes were retained and named in the litchi genome (**Supplementary Table S4**). The number of the *PP2C* family members from litchi surpasses that in grapes (27) (He et al., 2018), walnut (*Juglans regia*) (41) (Chen et al., 2022), and strawberry (62) (Haider et al., 2019, whereas which is lower than in rice (78) (Xue et al., 2008), Arabidopsis (80) (Xue et al., 2008), and apple (128) (Wang et al., 2021), connoting that the number of *PP2C* genes may has correlation with reasons such as genome size (Liu et al., 2022) or whole genome duplication (Zhang et al., 2015). The 68 LcPP2C proteins had theoretical pI values ranging from 4.60 (*LcPP2C32*) to 9.61 (*LcPP2C9*) and most had pI values < 7.00, which is similar to that obtained from identifications of turnip (*Brassica rapa*) (Khan et al., 2019) and paper mulberry (*Broussonetia papyrifera*) (Zhang et al., 2021a).

In terms of evolutionary relationships, the 68 *LcPP2C* members were clustered into thirteen groups with reference to the topology of phylogenetic tree and the classification of PP2C subfamilies in Arabidopsis and rice. The thirteen groups were designated A, B, C, D, E, F1, F2, G, H, I, J, K, and L using English letters (**Figure 1**), as employed for Arabidopsis and rice (Xue et al., 2008). Most of these groups are common and are similar to the classification reported in previous phylogenetic analyses in other plant species, such as maize (Fan et al., 2019), soybean (*Glycine max*) (Fan et al., 2020), and barley (*Hordeum vulgare*) (Wu et al., 2022). This finding showed that *PP2C* gene family of various species are highly conserved during evolution, and then suggested that the *PP2C* members belonging to the conserved groups among species may play fundamental roles during plant development and evolution. It is noteworthy that all groups contained *PP2C* members from litchi, Arabidopsis, and rice, but the multiple *LcPP2C* genes were tightly clustered with *AtPP2C* genes in the same group, indicating that these genes may have high homology and similar functions. Therefore, we confirmed that litchi and dicotyledonous Arabidopsis have a closer relationship compared to monocotyledonous rice. Strikingly, the 6 *LcPP2C* members (*LcPP2C8*/*9*/*21*/*39*/*50*/*61*) were not included in current relevant groups, just like several genes (*AtPP2C8*/*31*/*44*/*54*/*57*/*70* and *OsPP2C4*/*5*/*21*/*67*) among Arabidopsis and rice (Xue et al., 2008). It may be due to the increase of these members during the evolution, further proving that some unclassified genes may have specifically evolved to meet the developmental needs or the stress resistance in plants. In addition, a few of the adjacent *LcPP2C* genes contained on the same chromosome were clustered together in the phylogenetic tree; for example, *LcPP2C16*/*17*/*18* were classified into group A, *LcPP2C45*/*46* were classified into H, and *LcPP2C66*/*67*/*68* were classified into D, which indicated that these genes mentioned above have recent evolutionary origins and conserved molecular functions. Similar phenomena were also found in strawberry (Haider et al., 2019) and cucumber (*Cucumis sativus*) (Zhang et al., 2022b).

The variation in exon–intron structure is judged to be an important indicator of the origin and evolution among multigene families (Zhang et al., 2023). Ulteriorly, the greater the number of introns, the higher the recombination frequency the genes, which may affect the number of members between a gene family (Xiong et al., 2024). Our results showed that the number of introns among the *LcPP2C* genes varied from one to twenty (**Figure 2**), implying that intron gain or loss events were one of the main causes of the *LcPP2C* gene family expansion. It is supported by that identified in soybean (Fan et al., 2020) and *Dendrobium catenatum* (Zhang et al., 2021b). Conserved motif analysis of the *LcPP2C* family revealed fourteen motifs, and all the members were distributed with Motif 1/2 (**Figure 3**), demonstrating that Motif 1/2 may be the PP2C motifs of this family. As a whole, the *LcPP2C* members among the same group had similar gene structures and conserved motif distribution, while different groups had larger differences, showing that conservation within the same group and similar functions, which is analogous to the results from alfalfa (*Medicago truncatula*) (Yang et al., 2018) and cotton (*Gossypium hirsutum*) (Shazadee et al., 2019). Meanwhile, there were some exceptions; for example, *LcPP2C1* lost two introns and added Motif 13 relative to other genes among group G, and *LcPP2C41* lost one intron and reduced Motif 6 relative to other genes among F2, which may be due to events such as selection pressure or functional differentiation (Zhang, 2003; Rogozin et al., 2012).

Gene duplication events frequently occur in plants. In this way, plants can evolve with higher efficiency (Sun et al., 2023). There are five main types in which duplication formation were accomplished, namely whole genome duplication (WGD), transposed duplication (TRD), dispersed duplication (DSD), SD, and TD (Qiao et al., 2019; Wang et al., 2023a). SD and TD have been demonstrated to represent two of the main causes of duplicate gene production in plants (Cannon et al., 2004; Zhu et al., 2014). In this study, twelve SD events were found which occurred on eleven of fifteen chromosomes, and where the each *LcPP2C* gene presented 1 to 2 orthologous genes (**Figure 5**; **Supplementary Table S6**), indicating that SD was an important factor in the amplification of the LcPP2C family, which is consistent with the collinearity analyses of *Brachypodium distachyon* (Cao et al., 2016) and wheat (*Triticum aestivum*) (Yu et al., 2019). More than half of the members (*LcPP2C16*/*17*/*18*/*27*/*29*/*30*/*57*) among the group A in the *LcPP2C* family had SD events, uncovering that the expansion of the group A may be related to SD, which may also be one of the reasons why the number of members among the A was higher than that of other groups. The Ka/Ks ratios of 12 pairs of genes showed that purifying selection assumed primary responsibility of maintaining the functions of the *LcPP2C* genes, which is similar to the reports in strawberry (Haider et al., 2019) and paper mulberry (Zhang et al., 2021a). Moreover, the collinear relationships of the *LcPP2C* gene family and dicotyledons were found to be greater and less with monocotyledons (**Figure 6**; **Supplementary Tables S8–13**), which may be connected to the classification of dicotyledonous and monocotyledonous plants produced by angiosperms during long-term natural selection and evolution (Xiong et al., 2024).

As is known to all, cis-acting elements are key components of the gene regulatory network which combine with transcription factors (TFs) to regulate the transcription process in the target genes (Zhao et al., 2020). The gibberellin, abscisic acid, methyl jasmonate and salicylic acid have been shown to play important roles in mediating defense responses to abiotic stresses (Yang et al., 2013; Chen et al., 2016; Wang et al., 2015; Liu et al., 2016), and it has also been reported that these hormones mentioned above can target their corresponding elements (GARE-motif, P-box, TATC-box, ABRE, TGACG-motif, CGTCA-motif, and TCA-element) to regulate the expression of stress-related genes, which enables plants to better cope with adverse environment (Kummari et al., 2020). Additionally, distinct stress-related elements (MBS, LTR, ARE, GC-motif, and TC-rich repeats) among genes reflect their great potential in plant stress resistance (Yang et al., 2022). Therefore, we analyzed the cis-acting elements in the promoter regions of the *LcPP2C* genes. Here, abundant elements were implicated in hormones and stresses and were irregularly scattered in the promoter regions (**Figure 8**; **Supplementary Table S14**), connoting that various elements associated with different hormones and stresses may have contributed to the resistance effects of the *LcPP2C* genes, which is similar to that gained from predictions of Foxtail Millet (*Setaria italica*) (Min et al., 2013) and pear (*Pyrus bretschneideri*) (Wang et al., 2021). Among the LcPP2C family, 66 members (except *LcPP2C13*/*44*) had at least one of those four hormone-related (gibberellin, abscisic acid, methyl jasmonate, and salicylic acid) elements, while 67 members (except *LcPP2C39*) had at least one of those five stress-related (drought, low temperature, anaerobic induction, anoxic induction, and defense and stress) elements, suggesting that the *LcPP2C* family may be broadly involved in complex signaling pathway regulation to deal with adverse environmental conditions.

It is known that tissue-specific expression profiles of genes are significant for analyzing gene functions in plants (Zhang et al., 2023). Our study found that the exclusive expression of *LcPP2C30* in leaves of litchi (**Figure 9**; **Supplementary Table S15**), suggesting its role in phloem unloading. Meanwhile, the remaining 67 genes had distinct expression in different tissues, indicating that the differential functions of the *LcPP2C* family members, which validates the correctness of analytical deductions in phylogenetic relationships, gene structures, and cis-acting elements. Strikingly, the highest number of the *LcPP2C* genes (54) expressed significantly (with more than ten) in male flowers, proving that the *LcPP2C* genes may play major roles in differentiation and development of flower organs, which is similar to the finding of Fan et al. (2019) and Shen et al. (2023) Moreover, 43 of the 68 *LcPP2C* genes (except *LcPP2C5*/*6*/*9*/*15*/*20*/*21*/*23*/*24*/*30*/*32*/*38*/*39*/*40*/*48*/*49*/*50*/*51*/*53*/*60*/*61*/*62*/*64*/*65*/*67*/*68*) had predominant expression in roots, implying that the *LcPP2C* genes may be involved in root development and adaptive stress responses, which is supported by the results in poplar (*Populus trichocarpa*) (Rigoulot et al., 2019) and paper mulberry (Zhang et al., 2021a). Eventually, these data lay a foundation for exploring the functions of the *LcPP2C* family members.

A variety of *PP2C* genes have been reported to be associated with plant responses to abiotic stresses (Wang et al., 2023b). Accordingly, for further exploring degree of the *LcPP2C* genes response to diverse stresses (cold, heat, and drought), we picked out 63 members to detect their expression at different time after the treatments, combined with the analyses of cis-acting elements and tissue-specific expression. The results showed that the majority of the *LcPP2C* genes were induced but only a few of them were induced slightly by at least one of the three stress treatments (**Figure 10**). Similar expression status was found for *PP2C* genes in *Brachypodium distachyon* (Cao et al., 2016) and pear (Wang et al., 2021). Particularly, among these abiotic stress-induced *LcPP2C* genes, some exhibited rapidly and had increased to the highest level at 3 h after treatments, indicating that these genes may perform pioneering functions in resistance to abiotic stresses of litchi; by comparison, some expressed gradually and expression level peaked at 6 h or 12 h, suggesting that these genes played the biggest roles at the late stages of resisted stresses; and besides, the expression level of others rose to the highest level with the extension of treatment time, inferring that these genes had not finished their responses to stresses. Intriguingly, the expression level of a few of the *LcPP2C* genes were inhibited after at least one of the three treatments, connoting that these genes may have defense and other specific functions in litchi. Expectedly, nine pairs of SD genes (*LcPP2C4*/*38*, *LcPP2C16*/*17*, *LcPP2C16*/*29*, *LcPP2C17*/*29*, *LcPP2C18*/*30*, *LcPP2C22*/*55*, *LcPP2C27*/*57*, *LcPP2C34*/*53*, and *LcPP2C35*/*52*) expressed similar tendencies within adversities resistance process of litchi, despite their time points to reach the highest level were not exactly the same. Equally, the physiological and biochemical character of litchi seedlings showed that abiotic stresses may take the main responsibility for regulating the transcription and expression of the *LcPP2C* genes, which is consistent with the researches of rice (Singh et al., 2010) and tomato (*Solanum lycopersicum*) (Zhang et al., 2018). On the whole, the results show that the *LcPP2C* family members exhibited diverse expression patterns in different tissues, at different time, and under different stress treatments, demonstrating that the functional diversification of the *LcPP2C* gene family as well as its key roles in the tissue development and abiotic stress responses for litchi. The findings can provide candidate targets for the genetic improvement and establish a theoretical foundation for breeding excellent varieties in litchi.

## 5 Conclusion

In summary, a total of 68 *LcPP2C* genes were identified for the first time from the whole genome of litchi which were divided into thirteen groups (A, B, C, D, E, F1, F2, G, H, I, J, K, and L), and these groups exhibited similar gene structures and motif distribution. The 68 *LcPP2C* genes were randomly distributed across fourteen chromosomes, which had twelve pairs of segmental duplication events and had the highest homology with dicotyledonous plants. The cis-acting elements analysis revealed that the *LcPP2C* genes participated extensively in the growth and development as well as hormonal and stress responses of litchi. Additionally, the *LcPP2C* family was tissue-specific during growth and development and responded to abiotic stress signals. This study provides insights into the functions and biochemical mechanism of the *LcPP2C* gene family and lays a basis for future research on validating precise regulation of the *LcPP2C* genes.

## Data Availability

The datasets presented in this study can be found in online repositories. The names of the repository/repositories and accession number(s) can be found in the article/**Supplementary Material**.

## References

[B1] Ahn C. S. Lee D. H. Pai H. S. (2019). Characterization of Maf1 in *Arabidopsis*: function under stress conditions and regulation by the TOR signaling pathway. Planta. 249, 527–542. doi: 10.1007/s00425-018-3024-5 30293201

[B2] Bhaskara G. B. Wong M. M. Verslues P. E. (2019). The flip side of phospho-signalling: Regulation of protein dephosphorylation and the protein phosphatase 2Cs. Plant Cell E. 42, 2913–2930. doi: 10.1111/pce.13616 31314921

[B3] Cannon S. B. Mitra A. Baumgarten A. Young N. D. May G. (2004). The roles of segmental and tandem gene duplication in the evolution of large gene families in *Arabidopsis thaliana* . BMC Plant Biol. 4, 10. doi: 10.1186/1471-2229-4-10 15171794 PMC446195

[B4] Cao J. Jiang M. Li P. Chu Z. (2016). Genome-wide identification and evolutionary analyses of the *PP2C* gene family with their expression profiling in response to multiple stresses in *Brachypodium distachyon* . BMC Genomics 17, 175. doi: 10.1186/s12864-016-2526-4 26935448 PMC4776448

[B5] Castellanos R. U. Friedrich T. Petrovic N. Altmann S. Brzezinka K. Gorka M.. (2020). FORGETTER2 protein phosphatase and phospholipase D modulate heat stress memory in Arabidopsis. Plant J. 104, 7–17. doi: 10.1111/tpj.14927 32654320

[B6] Chen C. Wu Y. Li J. Wang X. Zeng Z. Xu J.. (2023). TBtools-II: A ‘‘one for all, all for one’’ bioinformatics platform for biological big-data mining. Mol. Plant 16, 1733–1742. doi: 10.1016/j.molp.2023.09.010 37740491

[B7] Chen H. Y. Hsieh E. J. Cheng M. C. Chen C. Y. Hwang S. Y. Lin T. P. (2016). ORA47 (octadecanoid-responsive AP2/ERF-domain transcription factor 47) regulates jasmonic acid and abscisic acid biosynthesis and signaling through binding to a novel *cis*-element. New Phytol. 211, 599–613. doi: 10.1111/nph.13914 26974851

[B8] Chen S. Deng J. Cheng P. Zhang Z. Wang Y. Chen S.. (2022). Transcriptome-wide identification of walnut *PP2C* family genes in response to external stimulus. BMC Genomics 23, 640. doi: 10.1186/s12864-022-08856-3 36076184 PMC9461273

[B9] Ding F. Zhang S. Chen H. Su Z. Zhang R. Xiao Q.. (2015). Promoter difference of *LcFT1* is a leading cause of natural variation of flowering timing in different litchi cultivars (*Litchi chinensis* Sonn.). Plant Sci. 241, 128–137. doi: 10.1016/j.plantsci.2015.10.004 26706065

[B10] Dubrovinaa A. S. Kiseleva K. V. Khristenkoa V. S. Aleynova O. A. (2015). *VaCPK20*, a calcium-dependent protein kinase gene of wild grapevine *Vitis amurensis* Rupr., mediates cold and drought stress tolerance. J. Plant Physiol. 185, 1–15. doi: 10.1016/j.jplph.2015.05.020 26264965

[B11] Fan K. Chen Y. Mao Z. Fang Y. Li Z. Lin W.. (2020). Pervasive duplication, biased molecular evolution and comprehensive functional analysis of the PP2C family in *Glycine max* . BMC Genomics 21, 465. doi: 10.1186/s12864-020-06877-4 32631220 PMC7339511

[B12] Fan K. Yuan S. Chen J. Chen Y. Li Z. Lin W.. (2019). Molecular evolution and lineage−specific expansion of the PP2C family in *Zea mays* . Planta. 250, 1521–1538. doi: 10.1007/s00425-019-03243-x 31346803

[B13] Finkelstein R. (2013). Abscisic acid synthesis and response. Arabidopsis Book. 1, e0166. doi: 10.1199/tab.0166 PMC3833200 24273463

[B14] Fuchs S. Grill E. Meskiene I. Schweighofer A. (2013). Type 2C protein phosphatases in plants. FEBS J. 280, 681–693. doi: 10.1111/j.1742-4658.2012.08670.x 22726910

[B15] Gong Z. Xiong L. Shi H. Yang S. Herrera-Estrella L. R. Xu G.. (2020). Plant abiotic stress response and nutrient use efficiency. Sci. China Life Sci. 63, 635–674. doi: 10.1007/s11427-020-1683-x 32246404

[B16] Guan H. Wang H. Huang J. Liu M. Chen T. Shan X.. (2021). Genome-Wide identification and expression analysis of MADS-box family genes in Litchi (*Litchi chinensis* Sonn.) and their involvement in floral sex determination. Plants. 10, 2142. doi: 10.3390/plants10102142 34685951 PMC8540616

[B17] Haider M. S. Khan N. Pervaiz T. Liu Z. Nasim M. Jogaiah S.. (2019). Genome-wide identification, evolution, and molecular characterization of the *PP2C* gene family in woodland strawberry. Gene. 702, 27–35. doi: 10.1016/j.gene.2019.03.025 30890476

[B18] He H. Lu Z. Ma Z. Liang G. Ma L. Wan P.. (2018). Genome-wide identification and expression analysis of the PP2C gene family in *Vitis vinifera* . Acta Hortic. Sinica. 45, 1237–1250. doi: 10.16420/j.issn.0513-353x.2017-0824

[B19] He Y. Liu Y. Li M. Lamin-Samu A. T. Yang D. Yu X.. (2021). The Arabidopsis SMALL AUXIN UP RNA32 protein regulates ABA-mediated responses to drought stress. Front. Genet. 12. doi: 10.3389/fpls.2021.625493 PMC7994887 33777065

[B20] Hu G. Feng J. Xiang X. Wang J. Salojärvi J. Liu C.. (2022). Two divergent haplotypes from a highly heterozygous lychee genome suggest independent domestication events for early and late-maturing cultivars. Nat. Genet. 54, 73–83. doi: 10.1038/s41588-021-00971-3 34980919 PMC8755541

[B21] Hu J. (2018). Litchi flowering is regulated by expression of *short Vegetative Phase* genes. J. Amer. Soc Hortic. Sci. 143, 101–109. doi: 10.21273/JASHS04316-17

[B22] Hu X. Liu L. Xiao B. Li D. Xing X. Kong X.. (2010). Enhanced tolerance to low temperature in tobacco by over-expression of a new maize protein phosphatase 2C, *ZmPP2C2* . J. Plant Physiol. 167, 1307–1315. doi: 10.1016/j.jplph.2010.04.014 20580122

[B23] Hulsmans S. Rodriguez M. Coninck B. D. Rolland F. (2016). The SnRK1 energy sensor in plant biotic interactions. Trends Plant Sci. 21, 648–661. doi: 10.1016/j.tplants.2016.04.008 27156455

[B24] Jung C. Nguyen N. H. Cheong J. (2020). Transcriptional regulation of protein phosphatase 2C genes to modulate abscisic acid signaling. Int. J. Mol. Sci. 21, 9517. doi: 10.3390/ijms21249517 33327661 PMC7765119

[B25] Khan N. Ke H. Hu C. M. Naseri E. Haider M. S. Ayaz A.. (2019). Genome-wide identification, evolution, and transcriptional profiling of *PP2C* gene family in *Brassica rapa* . BioMed. Res. Int. 2019, 15. doi: 10.1155/2019/2965035 PMC6470454 31073524

[B26] Komatsu K. Suzuki N. Kuwamura M. Nishikawa Y. Nakatani M. Ohtawa H.. (2013). Group A PP2Cs evolved in land plants as key regulators of intrinsic desiccation tolerance. Nat. Commun. 4, 2219. doi: 10.1038/ncomms3219 23900426 PMC3731658

[B27] Kummari D. Palakolanu S. R. Kishor P. B. K. Bhatnagar-Mathur P. Singam P. Vadez V.. (2020). An update and perspectives on the use of promoters in plant genetic engineering. J. Biosci. 45, 119. doi: 10.1007/s12038-020-00087-6 33097676

[B28] Li Y. S. Sun H. Wang Z. F. Duan M. Huang S. D. Yang J.. (2013). A novel nuclear protein phosphatase 2C negatively regulated by ABL1 is involved in abiotic stress and panicle development in rice. Mol. Biotechnol. 54, 703–710. doi: 10.1007/s12033-012-9614-8 23086454

[B29] Liu L. Sonbol F. M. Huot B. Gu Y. Withers J. Mwimba M.. (2016). Salicylic acid receptors activate jasmonic acid signalling through a non-canonical pathway to promote effector-triggered immunity. Nat. Commun. 7, 13099. doi: 10.1038/ncomms13099 27725643 PMC5062614

[B30] Liu Y. Liu X. Yang D. Yin Z. Jiang Y. Ling H.. (2022). A comprehensive identification and expression analysis of VQ motif-containing proteins in sugarcane (*Saccharum* sp*ontaneum* L.) under phytohormone treatment and cold stress. Int. J. Mol. Sci. 23, 6334. doi: 10.3390/ijms23116334 35683012 PMC9181594

[B31] Lv J. Liu J. Ming Y. Shi Y. Song C. Gong Z.. (2021). Reciprocal regulation between the negative regulator PP2CG1 phosphatase and the positive regulator OST1 kinase confers cold response in *Arabidopsis* . J. Integr. Plant Biol. 63, 1568–1587. doi: 10.1111/jipb.13100 33871153

[B32] Min D. H. Xue F. Y. Ma Y. N. Chen M. Xu Z. S. Li L. C.. (2013). Characteristics of PP2C gene family in foxtail millet (*Setaria italica*). Acta Agronomica Sinica. 39, 2135–2144. doi: 10.3724/SP.J.1006.2013.02135

[B33] Park S. Y. Fung P. Nishimura N. Jensen D. R. Fujii H. Zhao Y.. (2009). Abscisic acid inhibits PP2Cs via the PYR/PYL family of ABA-binding START proteins. Science. 324, 1068–1071. doi: 10.1126/science.1173041 19407142 PMC2827199

[B34] Qiao X. Li Q. Yin H. Qi K. Li L. Wang R.. (2019). Gene duplication and evolution in recurring polyploidization–diploidization cycles in plants. Genome Biol. 20, 38. doi: 10.1186/s13059-019-1650-2 30791939 PMC6383267

[B35] Rigoulot S. B. Petzold H. E. Williams S. P. Brunner A. M. Beers E. P. (2019). *Populus trichocarpa* clade A PP2C protein phosphatases: their stress−induced expression patterns, interactions in core abscisic acid signaling, and potential for regulation of growth and development. Plant Mol. Biol. 100, 303–317. doi: 10.1007/s11103-019-00861-7 30945147

[B36] Rogozin I. B. Carmel L. Csuros M. Koonin E. V. (2012). Origin and evolution of spliceosomal introns. Biol. Direct. 7, 11. doi: 10.1186/1745-6150-7-11 22507701 PMC3488318

[B37] Schweighofer A. Hirt H. Meskiene I. (2004). Plant PP2C phosphatases: emerging functions in stress signaling. Trends Plant Sci. 9, 236–243. doi: 10.1016/j.tplants.2004.03.007 15130549

[B38] Schweighofer A. Kazanaviciute V. Scheikl E. Teige M. Doczi R. Hirt H.. (2007). The PP2C-type phosphatase AP2C1, which negatively regulates MPK4 and MPK6, modulates innate immunity, jasmonic acid, and ethylene levels in *Arabidopsis* . Plant Cell. 19, 2213–2224. doi: 10.1105/tpc.106.049585 17630279 PMC1955703

[B39] Servet C. Benhamed M. Latrasse D. Kim W. Delarue M. Zhou D. X. (2008). Characterization of a phosphatase 2C protein as an interacting partner of the histone acetyltransferase GCN5 in *Arabidopsis* . Bba-Biomembranes. 1779, 376–382. doi: 10.1016/j.bbagrm.2008.04.007 18498779

[B40] Shazadee H. Khan N. Wang J. Wang C. Zeng J. Huang Z.. (2019). Identification and expression profiling of protein phosphatases (*PP2C*) gene family in *Gossypium hirsutum* L. Int. J. Mol. Sci. 20, 1395. doi: 10.3390/ijms20061395 30897702 PMC6471114

[B41] Shen Y. Zou J. Luo P. Shang W. Li Y. He S.. (2023). Genome-wide identification and abiotic stress response analysis of PP2C family genes in *Rosa chinensis* ‘Old Blush’. Acta Hortic. Sinica. 50, 2139–2156. doi: 10.16420/j.issn.0513-353x.2022-0752

[B42] Singh A. Giri J. Kapoor S. Tyagi A. K. Pandey G. K. (2010). Protein phosphatase complement in rice: genome-wide identification and transcriptional analysis under abiotic stress conditions and reproductive development. BMC Genomics 11, 435. Available at: http://www.biomedcentral.com/1471-2164/11/435. 20637108 10.1186/1471-2164-11-435 PMC3091634

[B43] Singh A. Pandey A. Srivastava A. K. Phan Tran L. S. Pandey G. K. (2015). Plant protein phosphatases 2C: from genomic diversity to functional multiplicity and importance in stress management. Crit. Rev. Biotechnol. 36, 1023–1035. doi: 10.3109/07388551.2015.1083941 26380928

[B44] Smoly I. Shemesh N. Ziv-Ukelson M. Ben-Zvi A. Yeger-Lotem E. (2017). An asymmetrically balanced organization of kinases versus phosphatases across eukaryotes determines their distinct impacts. PloS Comput. Biol. 13, e1005221. doi: 10.1371/journal.pcbi.1005221 28135269 PMC5279721

[B45] Song S. K. Lee M. M. Clark S. E. (2006). POL and PLL1 phosphatases are CLAVATA1 signaling intermediates required for *Arabidopsis* shoot and floral stem cells. Development. 133, 4691–4698. doi: 10.1242/dev.02652 17079273

[B46] Sun Y. Jia X. Yang Z. Fu Q. Yang H. Xu X. (2023). Genome-wide identification of PEBP gene family in *solanum lycopersicum* . Int. J. Mol. Sci. 24, 9185. doi: 10.3390/ijms24119185 37298136 PMC10252294

[B47] Umbrasaite J. Schweighofer A. Kazanaviciute V. Magyar Z. Ayatollahi Z. Unterwurzacher V.. (2010). MAPK phosphatase AP2C3 Induces ectopic proliferation of epidermal cells leading to stomata development in Arabidopsis. PloS One 5, e15357. doi: 10.1371/journal.pone.0015357 21203456 PMC3009721

[B48] Wang C. Lu W. He X. Wang F. Zhou Y. Guo X.. (2016). The cotton *Mitogen-Activated* Protein Kinase Kinase 3 functions in drought tolerance by regulating stomatal responses and root growth. Plant Cell Physiol. 57, 1629–1642. doi: 10.1093/pcp/pcw090 27335349

[B49] Wang G. Sun X. Guo Z. Joldersma D. Guo L. Qiao X.. (2021). Genome-wide identification and evolution of the *PP2C* gene family in eight rosaceae species and expression analysis under stress in *Pyrus bretschneideri* . Front. Genet. 12. doi: 10.3389/fgene.2021.770014 PMC8632025 34858482

[B50] Wang L. Jin P. Wang J. Jiang L. Shan T. Zheng Y. (2015). Methyl Jasmonate primed defense responses against *Penicillium expansum* in sweet cherry fruit. Plant Mol. Biol. Rep. 33, 1464–1471. doi: 10.1007/s11105-014-0844-8

[B51] Wang Y. Li Y. Zhou F. Zhang L. Gong J. Cheng C.. (2023a). Genome-wide characterization, phylogenetic and expression analysis of *Histone* gene family in cucumber (*Cucumis sativus* L.). Int. J. Biol. Macromol. 230, 123401. doi: 10.1016/j.ijbiomac.2023.123401 36702227

[B52] Wang Y. Xu C. Li G. Ding Z. Zheng S. (2023b). Research progress of protein phosphatases type 2C family in response to various stresses in plants. Plant Physiol. J. 59, 1463–4173. doi: 10.13592/j.cnki.ppj.300173

[B53] Wei K. Wang Y. Xie D. (2014). Identification and expression profile analysis of the protein kinase gene superfamily in maize development. Mol. Breeding. 33, 155–172. doi: 10.1007/s11032-013-9941-x

[B54] Wei Y. Hu F. Hu G. Li X. Huang X. Wang H. (2011). Differential expression of anthocyanin biosynthetic genes in relation to anthocyanin accumulation in the pericarp of *litchi chinensis* sonn. PloS One 6, e19455. doi: 10.1371/journal.pone.0019455 21559331 PMC3084873

[B55] Wong J. H. Klejchova M. Snipes S. A. Nagpal P. Bak G. Wang B.. (2021). SAUR proteins and PP2C.D phosphatases regulate H^+^ -ATPases and K^+^ channels to control stomatal movements. Plant Physiol. 185, 256–273. doi: 10.1093/plphys/kiaa023 33631805 PMC8133658

[B56] Wu X. T. Xiong Z. P. Chen K. X. Zhao G. R. Feng K. R. Li X. H.. (2022). Genome-wide identification and transcriptional expression profiles of *PP2C* in the barley (*Hordeum vulgare* L.) pan-genome. Genes. 13, 834. doi: 10.3390/genes13050834 35627219 PMC9140614

[B57] Wu L. Zu X. Zhang H. Wu L. Xi Z. Chen Y. (2015). Overexpression of *ZmMAPK1* enhances drought and heat stress in transgenic *Arabidopsis thaliana* . Plant Mol. Biol. 88, 429–443. doi: 10.1007/s11103-015-0333-y 26008677

[B58] Xiao Q. S. Su Z. X. Chen H. B. Shen J. Y. (2018). Genome-wide identification and involvement of litchi *SPL* genes in flowering in response to cold and leaf maturity. J. Hortic. Sci. Biotech. 94, 428–440. doi: 10.1080/14620316.2018.1543557

[B59] Xiong R. Peng Z. Zhou H. Xue G. He A. Yao X.. (2024). Genome-wide identification, structural characterization and gene expression analysis of the WRKY transcription factor family in pea (*Pisum sativum* L.). BMC Plant Biol. 24, 113. doi: 10.1186/s12870-024-04774-6 38365619 PMC10870581

[B60] Xue T. Wang D. Zhang S. Ehlting J. Ni F. Jakab S.. (2008). Genome-wide and expression analysis of protein phosphatase 2C in rice and Arabidopsis. BMC Genomics 9, 550. doi: 10.1186/1471-2164-9-550 19021904 PMC2612031

[B61] Yang D. Dong X. Zhang Y. He Z. (2013). Gibberellins modulate abiotic stress tolerance in plants. Scientia Sin. Vitae. 43, 1119–1126. doi: 10.1360/052013-321

[B62] Yang J. Chen R. Liu W. Xiang X. Fan C. (2024a). Genome-wide characterization and phylogenetic and stress response expression analysis of the MADS-box gene family in litchi (*Litchi chinensis* Sonn.). Int. J. Mol. Sci. 25, 1754. doi: 10.3390/ijms25031754 38339030 PMC10855657

[B63] Yang J. Chen R. Liu W. Xiang X. Fan C. (2024b). Genome-wide identification and expression analysis of the Class III Peroxidase gene family under abiotic stresses in litchi (*Litchi chinensis* Sonn.). Int. J. Mol. Sci. 25, 5804. doi: 10.3390/ijms25115804 38891992 PMC11172018

[B64] Yang Q. Liu K. Niu X. Wang Q. Wan Y. Yang F.. (2018). Genome-wide identification of PP2C genes and their expression profiling in response to drought and cold stresses in *Medicago truncatula* . Sci. Rep-Uk. 8, 12841. doi: 10.1038/s41598-018-29627-9 PMC6110720 30150630

[B65] Yang T. Zhang P. Pan J. Amanullah S. Luan F. Han W.. (2022). Genome-wide analysis of the peroxidase gene family and verification of lignin synthesis-related genes in watermelon. Int. J. Mol. Sci. 23, 642. doi: 10.3390/ijms23020642 35054827 PMC8775647

[B66] Yu X. Han J. Wang E. Xiao J. Hu R. Yang G.. (2019). Genome-wide identification and homoeologous expression analysis of *PP2C* genes in wheat (*Triticum aestivum* L.). Front. Genet. 10. doi: 10.3389/fgene.2019.00561 PMC6582248 31249596

[B67] Zhang B. Chen N. Peng X. Shen S. (2021). Identification of the *PP2C* gene family in paper mulberry (*Broussonetia papyrifera*) and its roles in the regulation mechanism of the response to cold stress. Biotechnol. Lett. 43, 1089–1102. doi: 10.1007/s10529-021-03110-4 33751277

[B68] Zhang G. Wang F. Li J. Ding Q. Zhang Y. Li H.. (2015). Genome-wide identification and analysis of the VQ motif-containing protein family in Chinese cabbage (*Brassica rapa* L. ssp. *Pekinensis*). Int. J. Mol. Sci. 16, 28683–28704. doi: 10.3390/ijms161226127 26633387 PMC4691074

[B69] Zhang G. Zhang Z. Luo S. Li X. Lyu J. Liu Z.. (2022). Genome-wide identification and expression analysis of the cucumber *PP2C* gene family. BMC Genomics 23, 563. doi: 10.1186/s12864-022-08734-y 35933381 PMC9356470

[B70] Zhang H. Zhu J. Gong Z. Zhu J. K. (2022). Abiotic stress responses in plants. Genetics. 23, 104–119. doi: 10.1038/s41576-021-00413-0 34561623

[B71] Zhang J. (2003). Evolution by gene duplication: an update. Trends Ecol. Evol. 18, 292–298. doi: 10.1016/S0169-5347(03)00033-8

[B72] Zhang L. H. Zhu L. C. Xu Y. Lü L. Li X. G. Li W. H.. (2023). Genome-wide identification and function analysis of the sucrose phosphate synthase *MdSPS* gene family in apple. J. Integr. Agr. 22, 2080–2093. doi: 10.1016/j.jia.2023.05.024

[B73] Zhang T. Li Y. Zhang D. Kang Y. Wang J. Song X.. (2021). Genome-wide identification and expression analyses of *PP2C* gene family in *Dendrobium catenatum* . Acta Hortic. Sinica. 48, 2458–2470. doi: 10.16420/j.issn.0513-353x.2021-0382

[B74] Zhang Y. Li Q. Jiang L. Kai W. Liang B. Wang J.. (2018). Suppressing Type 2C protein phosphatases alters fruit ripening and the stress response in tomato. Plant Cell Physiol. 59, 142–154. doi: 10.1093/pcp/pcx169 29121241

[B75] Zhao Y. Zhao H. Wang Y. Zhang X. Zhao X. Yuan Z. (2020). Genome-wide identification and expression analysis of MIKC-type MADS-box gene family in *Punica granatum* L. Agronomy. 10, 1197. doi: 10.3390/agronomy10081197

[B76] Zhu Y. Wu N. Song W. Yin G. Qin Y. Yan Y.. (2014). Soybean (*Glycine max*) expansin gene superfamily origins: segmental and tandem duplication events followed by divergent selection among subfamilies. BMC Plant Biol. 14, 93. doi: 10.1186/1471-2229-14-93 24720629 PMC4021193

